# Relationships of eating behaviors with psychopathology, brain maturation and genetic risk for obesity in an adolescent cohort study

**DOI:** 10.1038/s44220-024-00354-7

**Published:** 2025-01-10

**Authors:** Xinyang Yu, Zuo Zhang, Moritz Herle, Tobias Banaschewski, Gareth J. Barker, Arun L. W. Bokde, Herta Flor, Antoine Grigis, Hugh Garavan, Penny Gowland, Andreas Heinz, Rüdiger Brühl, Jean-Luc Martinot, Marie-Laure Paillère Martinot, Eric Artiges, Frauke Nees, Dimitri Papadopoulos Orfanos, Hervé Lemaître, Tomáš Paus, Luise Poustka, Sarah Hohmann, Nathalie Holz, Christian Bäuchl, Michael N. Smolka, Nilakshi Vaidya, Henrik Walter, Robert Whelan, Ulrike Schmidt, Gunter Schumann, Sylvane Desrivières, on behalf of the IMAGEN consortium, Tobias Banaschewski, Gareth J. Barker, Arun L. W. Bokde, Herta Flor, Antoine Grigis, Hugh Garavan, Penny Gowland, Andreas Heinz, Jean-Luc Martinot, Marie-Laure Paillère Martinot, Frauke Nees, Dimitri Papadopoulos Orfanos, Tomáš Paus, Luise Poustka, Michael N. Smolka, Henrik Walter, Robert Whelan, Gunter Schumann, Sylvane Desrivières, Uli Bromberg, Christian Büchel, Bernd Ittermann, Juliane H. Fröhner

**Affiliations:** 1 Social, Genetic and Developmental Psychiatry Centre, Institute of Psychiatry, Psychology and Neuroscience, King’s College London, London, UK; 2 Department of Child and Adolescent Psychiatry and Psychotherapy, Central Institute of Mental Health, Medical Faculty Mannheim, Heidelberg University, Mannheim, Germany; 3 Department of Neuroimaging, Institute of Psychiatry, Psychology and Neuroscience, King’s College London, London, UK; 4 Discipline of Psychiatry, School of Medicine and Trinity College Institute of Neuroscience, Trinity College Dublin, Dublin, Ireland; 5 Institute of Cognitive and Clinical Neuroscience, Central Institute of Mental Health, Medical Faculty Mannheim, Heidelberg University, Mannheim, Germany; 6 Department of Psychology, School of Social Sciences, University of Mannheim, Mannheim, Germany; 7 NeuroSpin, CEA, Université Paris-Saclay, Gif-sur-Yvette, France; 8 Departments of Psychiatry and Psychology, University of Vermont, Burlington, VT USA; 9 Sir Peter Mansfield Imaging Centre School of Physics and Astronomy, University of Nottingham, Nottingham, UK; 10 Charité – Universitätsmedizin Berlin, corporate member of Freie Universität Berlin, Humboldt-Universität zu Berlin, and Berlin Institute of Health, Department of Psychiatry and Psychotherapy, Campus Charité Mitte, Berlin, Germany; 11 Physikalisch-Technische Bundesanstalt (PTB), Berlin, Germany; 12 Institut National de la Santé et de la Recherche Médicale, INSERM U1299 ‘Developmental trajectories & psychiatry’, Université Paris-Saclay, Université Paris Cité, Ecole Normale supérieure Paris-Saclay, CNRS, Centre Borelli UMR9010, Gif-sur-Yvette, France; 13 Department of Child and Adolescent Psychiatry, AP-HP, Sorbonne Université, Pitié-Salpêtrière Hospital, Paris, France; 14 Institute of Medical Psychology and Medical Sociology, University Medical Center Schleswig Holstein, Kiel University, Kiel, Germany; 15 Institut des Maladies Neurodégénératives, UMR 5293, CNRS, CEA, Université de Bordeaux, Bordeaux, France; 16 Departments of Psychiatry and Neuroscience, Faculty of Medicine and Centre Hosptalier Universitaire Sainte-Justine, University of Montreal, Montreal, Quebec Canada; 17 Department of Child and Adolescent Psychiatry and Psychotherapy, University Medical Centre Göttingen, Göttingen, Germany; 18 Department of Child and Adolescent Psychiatry, Psychotherapy and Psychosomatics, University Medical Center Hamburg-Eppendorf, Hamburg, Germany; 19 Department of Psychiatry and Psychotherapy, Technische Universität Dresden, Dresden, Germany; 20 Centre for Population Neuroscience and Stratified Medicine (PONS), Department of Psychiatry and Psychotherapy, Charité Universitätsmedizin Berlin, Berlin, Germany; 21 School of Psychology and Global Brain Health Institute, Trinity College Dublin, Dublin, Ireland; 22 Department of Psychological Medicine, Centre for Research in Eating and Weight Disorders, Institute of Psychiatry, Psychology and Neuroscience, King’s College London, London, UK; 23 South London and Maudsley NHS Foundation Trust, London, UK; 24 Centre for Population Neuroscience and Stratified Medicine (PONS), Department of Psychiatry and Neuroscience, Charité Universitätsmedizin Berlin, Berlin, Germany; 25 Centre for Population Neuroscience and Precision Medicine (PONS), Institute for Science and Technology of Brain-inspired Intelligence (ISTBI), Fudan University, Shanghai, China; 26 University Medical Centre Hamburg-Eppendorf, Hamburg, Germany; 27 Department of Psychiatry and Neuroimaging Center, Technische Universität Dresden, Dresden, Germany

**Keywords:** Predictive markers, Risk factors

## Abstract

Unhealthy eating, a risk factor for eating disorders (EDs) and obesity, often coexists with emotional and behavioral problems; however, the underlying neurobiological mechanisms are poorly understood. Analyzing data from the longitudinal IMAGEN adolescent cohort, we investigated associations between eating behaviors, genetic predispositions for high body mass index (BMI) using polygenic scores (PGSs), and trajectories (ages 14–23 years) of ED-related psychopathology and brain maturation. Clustering analyses at age 23 years (*N* = 996) identified 3 eating groups: restrictive, emotional/uncontrolled and healthy eaters. BMI PGS, trajectories of ED symptoms, internalizing and externalizing problems, and brain maturation distinguished these groups. Decreasing volumes and thickness in several brain regions were less pronounced in restrictive and emotional/uncontrolled eaters. Smaller cerebellar volume reductions uniquely mediated the effects of BMI PGS on restrictive eating, whereas smaller volumetric reductions across multiple brain regions mediated the relationship between elevated externalizing problems and emotional/uncontrolled eating, independently of BMI. These findings shed light on distinct contributions of genetic risk, protracted brain maturation and behaviors in ED symptomatology.

## Abstract (web summary)

This study identifies distinct eating behavior profiles and links them to eating disorder symptoms, genetic predispositions for high body mass index and brain maturation during adolescence.

## Main

Eating disorders (EDs) are serious psychiatric disorders with high mortality rates, substantial impacts on quality of life and economic burdens^1,2^. Their increasing prevalence^3,4^, particularly during mid-adolescence^5^, highlights the need for early detection and effective interventions.

Key risk factors for EDs include eating behaviors such as dietary restraint and overeating, which increase the risk for unhealthy weight control behaviors and EDs like bulimia nervosa and binge ED^6^. Assessments of eating behaviors in various population groups^7,8^ indicated that cognitive restraint (CR), the conscious restriction of food intake to control body weight and shape, can lead to episodic overeating and is a strong predictor of disordered eating and negative body image. Conversely, uncontrolled eating (UE) (which refers to eating in response to food palatability, social cues and hunger, resulting in eating episodes) and emotional eating (EE) (eating episodes elicited by negative affect) is associated with increased susceptibility to EDs, hedonically driven food choices, higher body mass index (BMI) and obesity. Genetic studies indicate that these behaviors may be partially genetically determined, with obesity-associated variants linked to CR, UE, EE^9^, and eating behavior trajectories in childhood^10^ and adolescence^11^.

Neural factors also play a role in EDs^12^, with neurobiological differences observed in clinical samples^13–17^. Neurobehavioral correlates suggest that the hypothalamic, emotion/memory and executive systems are involved in eating control^18,19^. Neural activation of lateral prefrontal structures underpinning self-control and decision-making and striatal reward regions has been shown to underlie individual differences in CR^20^. Longitudinal studies have revealed volumetric brain differences, particularly in striatal and prefrontal regions, which suggest that differences in brain maturation may be etiological factors for disordered eating behaviors and comorbid depressive symptoms^21^.

Internalizing and externalizing psychopathology symptoms may serve as premorbid risk factors for EDs^22–24^. Externalizing problems (EPs) in early adolescence predict the onset^11^ and persistence of eating pathology^25^, whereas generalized anxiety symptoms predict adolescent-onset ED^26^. Negative affect and functional impairment are found to predict the onset of all EDs^6,27^. However, how trajectories of adolescent maladjustment, as evidenced by internalizing problems (IPs) and EPs, relate to eating behaviors, genetic liability and brain maturation is not well understood.

**Fig. 1:**
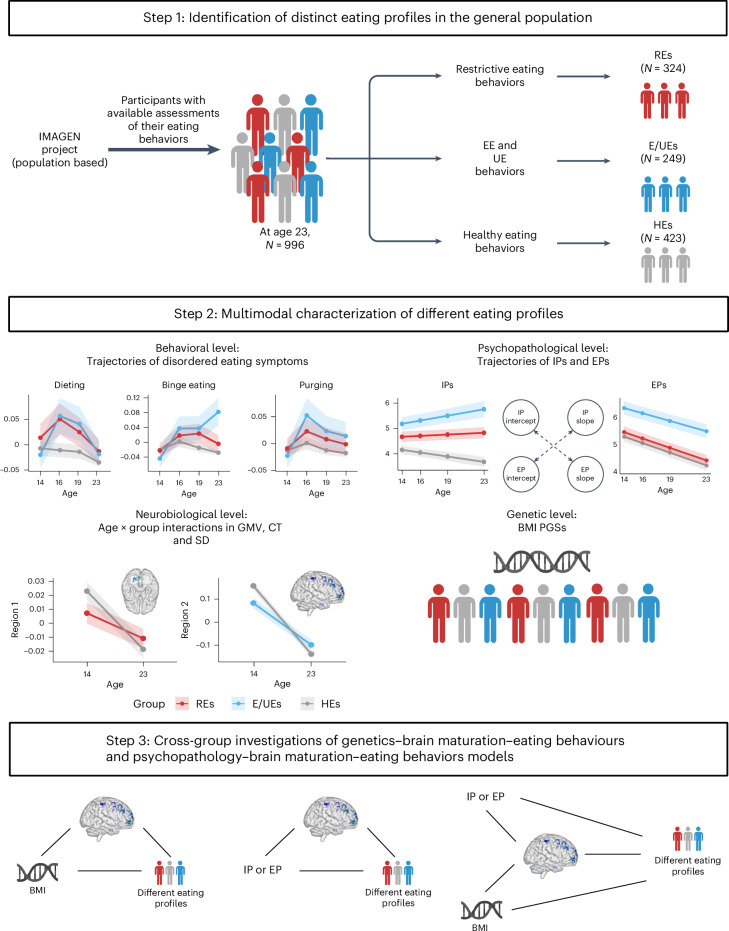
Workflow of research questions and analyses. EE, emotional eating; UE, uncontrolled eating; RE, restrictive eaters; E/UE, emotional and uncontrolled eaters; HE, healthy eaters; IP, internalizing problem; EP, externalizing problem; GMV, gray matter volume; CT, cortical thickness; SD, sulcal depth; BMI, body mass index; PGS, polygenic score. Regions 1 and 2 represent brain areas that showed a significant age-by-group interaction in their trajectory comparisons.

This study aimed to advance our understanding of eating behaviors by analyzing longitudinal data from the IMAGEN adolescent cohort. Applying a multivariate analytical framework, the study identified eating profiles at age 23, characterized by associations with polygenic scores (PGSs) for higher BMI, and differences in earlier trajectories of disordered eating, IPs and EPs, and brain maturation. The mediating roles of brain maturation and BMI PGS were also investigated using multivariate models. The analytical workflow is illustrated in Fig. 1.

## Results

### Identification of groups with distinct eating profiles

**Fig. 2:**
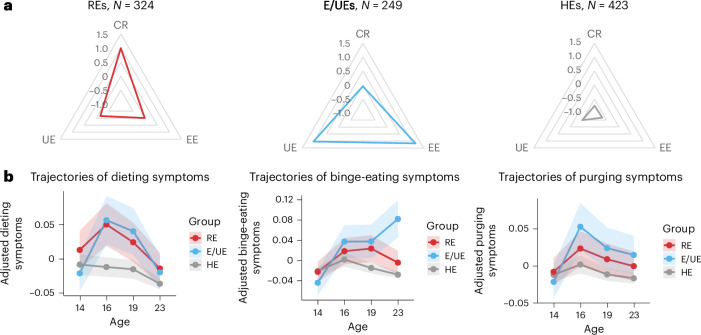
Distinct eating behavior profiles and their trajectories of ED symptoms across adolescence. **a**, Three groups of distinct eating behavior profiles were identified by *K*-means clustering at age 23. The radar charts show the average standardized scores for CR, EE and UE across these groups. **b**, Trajectories of ED symptoms (dieting, binge eating and purging symptoms) from ages 14 to 23 across the 3 identified groups. Data are presented as mean values ± 95% CIs. The points along the lines represent the mean symptom scores at each time, and the shaded areas show the 95% CIs. Analyses were adjusted for sex and recruitment sites. The *y* axis indicates the adjusted symptom scores after regressing out the effects of sex and recruitment sites.

A total of 996 participants (478 male and 518 female participants) with completed Three-Factor Eating Questionnaire (TFEQ) scores at age 23 and who had at least 1 measure from the Strengths and Difficulties Questionnaire (SDQ) available at ages 14, 16, 19 and 23 were included in this study (Methods). Three groups were identified from *K*-means clustering analysis with distinct eating behaviors (Fig. 2a and Table 1). The group distribution and within-group sum of squares are detailed in Supplementary Fig. 1. Validity and stability analyses confirmed the 3-group solution with Jaccard similarities of 0.83, 0.77 and 0.75, respectively. One group (*N* = 423) scored low on all eating behaviors, indicating healthy eaters (HEs). Another group (*N* = 324) showed the highest CR, indicating restrictive eaters (REs). Behaviors that differentiated REs the most from HEs (odds ratios > 5) included consciously eating less to control weight and weight gain, the intensity of restrained eating, consciously eating less than desired and not eating foods that made them fat (Supplementary Information). This group also scored significantly higher than HEs on UE. The 3rd group (*N* = 249) showed the highest EE and UE, indicating emotional and uncontrolled eaters (E/UEs). Behaviors that distinguished E/UEs the most from HEs included eating/overeating when feeling blue, lonely or anxious (all EE items), inability to stop eating and frequency of binge-eating episodes (UE items). This group also reported significantly higher CR than HEs. REs and E/UEs comprised predominantly female participants, contrasting with HEs, which had a higher proportion of male participants. Consistent with differences in BMI, the BMI PGSs were higher in REs and E/UEs than in HEs (Table 1).

**Table 1:** Sample sizes and demographic characteristics of the 3 groups of participants with distinct eating profiles at age 23

	REs (*N* = 324)	E/UEs (*N* = 249)	HEs (*N* = 423)	*F*/*χ*^*2*^	*P*	Post hoc tests (Bonferroni corrected, two sided)
Age at data collection, mean (s.d.)						
Baseline	14.56 (0.43)	14.49 (0.41)	14.49 (0.42)	4.93	0.027	NS
Follow-up 1	16.46 (1.15)	16.26 (1.66)	16.44 (1.39)	0.00	0.997	–
Follow-up 2	19.37 (0.95)	19.29 (1.01)	19.30 (0.98)	0.70	0.403	–
Follow-up 3	22.75 (0.74)	22.68 (0.72)	22.66 (0.73)	2.81	0.094	–
* N* male/female (% female participants)	133/191 (58.95%)	92/157 (63.05%)	253/170 (40.19%)	42.10	<0.001	REs versus HEs, *P* < 0.001; E/UEs versus HEs, *P* < 0.001
Eating behaviors, mean (s.d.)						
CR	17.55 (3.03)	12.90 (3.70)	9.78 (2.42)	623.94	<0.001	REs > E/UEs, *P* < 0.001; REs > HEs, *P* < 0.001; E/UEs > HEs, *P* < 0.001
EE	5.47 (1.76)	8.34 (2.13)	3.87 (1.22)	567.89	<0.001	E/UEs > REs, *P* < 0.001; E/UEs > HEs, *P* < 0.001; REs > HEs, *P* < 0.001
UE	18.84 (3.53)	25.15 (4.25)	17.06 (4.52)	307.45	<0.001	E/UEs > REs, *P* < 0.001; E/UEs > HEs, *P* < 0.001; REs > HEs, *P* < 0.001
Developmental stage, mean (s.d.)						
Pubertal status at age 14	3.03 (0.53)	2.99 (0.53)	2.79 (0.58)	34.47	<0.001	REs > HEs, *P* < 0.001; E/UEs > HEs, *P* < 0.001
Cognition, mean (s.d.)						
IQ^a^	109.91 (12.51)	108.51 (12.93)	112.12 (12.85)	6.04	0.014	E/UEs < HEs, *P* = 0.0018
EA	2.78 (1.32)	3.02 (1.62)	2.66 (1.18)	1.88	0.170	–
Anthropometric variable						
BMI *Z*-score at age 14, mean (s.d.)^b^	0.43 (0.77)	0.27 (0.90)	−0.17 (0.93)	83.34	<0.001	REs > HEs, *P* < 0.001; E/UEs > HEs, *P* < 0.001
Available MRI data for longitudinal MRI analysis, *N* (%)^c^	306 (94.44%)	236 (94.78%)	407 (96.22%)			
PGSs						
Available genotyping data of European ancestry, *N* (%)	285 (87.96%)	220 (88.35%)	376 (88.89%)			
BMI PGS *Z*-score, mean (s.d.)^d^	0.03 (1.02)	0.08 (0.88)	−0.24 (0.96)	13.56	<0.001	REs > HEs, *P* = 0.001; E/UEs > HEs, *P* < 0.001

*F-*tests were conducted for all variables except sex, for which chi-squared (*χ*^2^) tests were used to compare group differences. NS, not significant.

^a^IQ was calculated as the average of the PRI and VCI scores based on age norms using the WISC-IV.

^b^The age- and sex-adjusted BMI *Z*-score was calculated using the jBMI R package (https://github.com/jbirstler/jBmi) based on the Centers for Disease Control and Prevention recommendations.

^c^Participants were excluded from the analysis if they had missing MRI data or failed to meet QC criteria (*N* = 47; see Methods for image preprocessing and QC).

^d^Participants who passed genotyping QC (Supplementary Information) and were identified to be of European ancestry were selected for generating the BMI PGS. The BMI PGS was calculated using the publicly available PRScs and 1000 Genome Project 3 European LD panels (https://github.com/getian107/PRScs) and adjusted for the first 10 principal components of genetic ancestry and batch effects. The scores were *Z*-scored before analysis.

### Group differences in ED symptom trajectories in adolescence

Linear mixed models were applied to investigate group differences in trajectories of ED symptoms (dieting, binge eating and purging) from ages 14 to 23. Analyses of age-by-group interactions were used to identify symptom trajectories that differed in the RE or E/UE groups compared with HEs (Fig. 2b and Supplementary Tables 1 and 2). REs were characterized by significantly (*P* = 0.006) higher overall levels of dieting compared with HEs, with no significant age-by-group interactions. Trends toward increased dieting from ages 14 to 16 and increased binge eating from ages 14 to 19 were observed in this group. In E/UEs, significant age-by-group interactions were observed compared with HEs, with significantly increased dieting from ages 14 to 16 (*P*_Bonferroni_ = 0.026), and increased binge eating from ages 14 to 19 (*P*_Bonferroni_ = 0.028) and 14 to 23 (*P*_Bonferroni_ < 0.001). Nominal increases in purging were observed from ages 14 to 16, ages 14 and 19, and ages 14 to 23.

### IP and EP trajectories by group

**Fig. 3:**
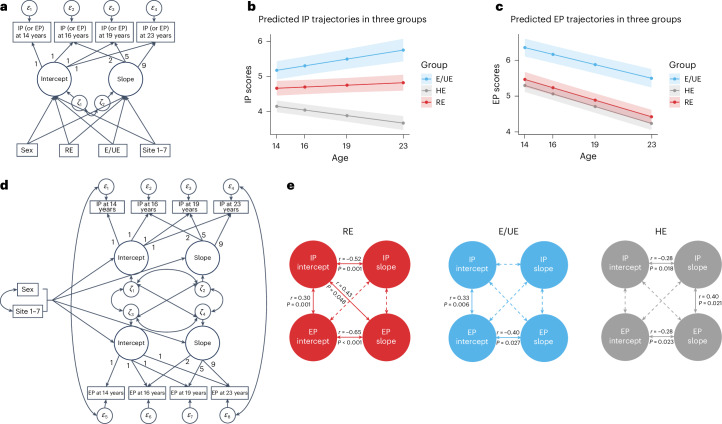
Psychopathological characterization across groups using IPs and EPs. **a**, Path diagram for the conditional linear LGCM for individual IP and EP trajectories among three groups. Two dummy variables, RE and E/UE, were included to represent three groups (HE was considered the reference group in the model). Sex and seven dummy variables for different recruitment sites were included as covariates in the analysis. Post hoc analyses were conducted to examine the differences between the RE and E/UE groups. **b**, Predicted trajectories of IP across three groups. **c**, Predicted trajectories of EP across three groups. Data are presented as mean values ± 95% CIs. Points along the lines represent the mean IP or EP scores at each time, and the shaded areas show the 95% CIs. **d**, Path diagram for the multivariate LGCM for each group separately. **e**, Significant within-construct and cross-construct correlations (that is, covariances among the latent factors) between IP and EP trajectories were found within each group. The significant (two-sided) standardized covariances (that is, correlation coefficient *r*) and *P* values are indicated in the figure. In the path diagrams in panels **a** and **d,** *ε* represents residuals for observed indicator variables (for example, IP or EP at each time point) after accounting for the influence of latent factors (intercept and slope); *ζ* represents residuals for latent variables, capturing vairance that is not explained by the predictors. The numbers 1, 2, 5 and 9 indicate the factor loadings for the intercept and slope, reflecting the time intervals between the observed indicator variables.

We explored behavioral group differences further, using latent growth curve models (LGCMs) to measure trajectories of IPs and EPs across (Fig. 3a; for descriptives, see Supplementary Table 1 and Supplementary Fig. 3) and within groups (Fig. 3d). Between-group univariate analyses showed that, compared with HEs, unhealthy eaters (REs and E/UEs) showed significant differences in how their IPs developed over time. Both REs (*β* = 0.073, 95% confidence interval (CI) = 0.019–0.126, *P* = 0.008; Fig. 3b) and E/UEs (*β* = 0.110, 95% CI = 0.051–0.169, *P* < 0.001) reported a significant increase (that is, in the slopes of their trajectories; Supplementary Tables 3 and 4) in IPs with age. Intercepts of IPs also differed, with E/UEs already reporting higher levels of IPs at age 14 than HEs (*β* = 0.712, 95% CI = 0.281–1.144, *P* = 0.001). Regarding EP trajectories, all three groups showed a decrease in these problems over time but the rate of decrease did not significantly differ between groups (Fig. 3c). Nevertheless, the EPs reported at age 14 were higher in E/UEs compared with HEs (*β* = 0.855, 95% CI = 0.418–1.292, *P* < 0.001) and REs (*β* = 0.743, 95% CI = 0.278–1.209, *P* = 0.002).

Within-group multivariate LGCM analyses, which included IPs and EPs in the same model (Fig. 3d), revealed significant within- and between-construct correlations (Fig. 3e). Within-construct correlations showed that, in all groups, higher levels of EPs at age 14 were significantly correlated with smaller decreases in these problems over time. This association was especially strong in unhealthy eaters (REs and E/UEs), indicating that higher initial levels of EPs were linked to less improvement over time. Similarly, in REs and HEs, higher initial levels of IPs were associated with smaller decreases in these problems over time, but this pattern was not observed in E/UEs. Interestingly, our models highlighted notable connections between IPs and EPs. Specifically, we found that these problems tended to co-occur, especially in unhealthy eaters (REs and E/UEs), suggesting that individuals who started with higher levels of one type of problem were also more likely to have higher levels of the other. In HEs, changes in IPs and EPs were positively correlated, indicating that as one type of problem decreased, the other also tended to decrease. In addition, in contrast to the other groups, in REs there was a significant positive correlation between the initial levels of IPs and the changes in EPs over time, suggesting that higher initial levels of IPs were related to more subsequent changes in EPs. No other significant relationships between IPs and EPs were found in these analyses.

### Group differences in brain maturation across adolescence

Longitudinal analyses were conducted to investigate between-group differences in brain maturation during adolescence, comparing changes in gray matter volume (GMV), cortical thickness (CT) and sulcal depth (SD) from age 14 to 23. All primary analyses included sex, recruitment sites and total intracranial volume (TIV) as covariates.

**Fig. 4:**
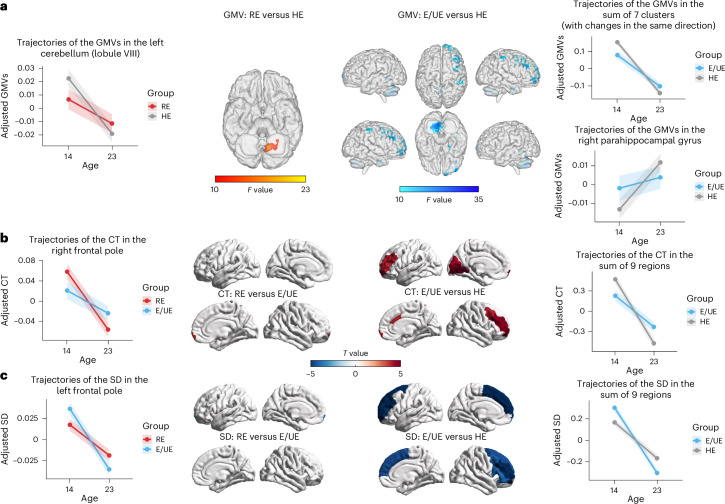
Significant age-by-group interactions were identified in various brain morphological features, including GMV, CT and SD. **a**, A significant age-by-group interaction was observed between REs and HEs, ages 14–23, indicating that REs experienced a smaller volumetric reduction in the left cerebellum compared with HEs over time. E/UEs showed less volumetric reductions in multiple brain regions, including the left cerebellum, right MFG, right medial superior frontal gyrus, right postcentral gyrus and right putamen compared with HEs. However, E/UEs had volumetric increases in the right parahippocampal gyrus compared with HEs. **b**, Comparisons of CT trajectories between REs and E/UEs showed that REs experienced more pronounced CT reductions in the right frontal pole. CT trajectory comparisons between HEs and E/UEs showed that HEs experienced more pronounced CT reductions across a wide range of brain regions, including the frontal and occipital gyri and the anterior cingulate gyrus compared with E/UEs. **c**, SD trajectory comparisons between REs and E/UEs indicated that REs had less pronounced SD reductions in the left frontal pole. Comparisons of SD trajectories between HEs and E/UEs showed that HEs had less pronounced SD reductions in the frontal and temporal regions. All analyses were adjusted for sex, recruitment sites and TIV. The lines in the figures represent mean values for brain morphological features and the shaded areas represent the corresponding 95% CIs. The *y* axis indicates the adjusted brain morphological measures (GMV, CT or SD) after regressing out sex, recruitment sites and TIV. The *F* and *T* values were obtained from the 2 × 2 mixed analysis of variance and linear mixed model analyses, respectively (see Methods).

For GMV (Fig. 4a and Supplementary Table 6), longitudinal voxel-based morphometry (VBM) analyses were conducted, which revealed significant age-by-group interactions. Compared with HEs, REs showed smaller GMV reductions in the left cerebellum. E/UEs had smaller GMV reductions in two subclusters in the left cerebellum and five subclusters in the right hemisphere, including the middle frontal gyrus (MFG), putamen, medial superior frontal gyrus and postcentral gyrus. A lower GMV increase in the right parahippocampal gyrus was also observed. Differences remained similar after controlling for the joint effects of pubertal status, IQ, educational attainment (EA), and age- and sex-adjusted BMI (Supplementary Table 7). No significant differences in GMV trajectory were observed between REs and E/UEs.

For CT (Fig. 4b and Supplementary Table 8), mixed linear analyses showed significant age-by-group interactions in the right frontal pole when comparing E/UEs with REs, with REs experiencing more pronounced CT reduction. In contrast, comparing E/UEs with HEs showed significant interactions across nine brain regions. These included the left lingual gyrus, bilateral frontal pole, bilateral rostral MFG, left pericalcarine, left cuneus, right caudal MFG and right caudal anterior cingulate gyrus, with E/UEs showing less CT reduction than HEs. Most differences remained significant after controlling for the joint effects of pubertal status, IQ, EA, age- and sex-adjusted BMI, Euler’s number and their joint effects (Supplementary Table 9).

Regarding SD (Fig. 4c and Supplementary Table 10), REs had a less pronounced reduction in the left frontal pole compared with E/UEs. Moreover, E/UEs showed larger SD reductions across nine regions compared with HEs, including the bilateral rostral MFG, left frontal pole, bilateral superior frontal gyrus, right caudal MFG, right pars orbitalis, right pars opercularis and right pars triangularis gyrus. Most differences remained significant after controlling for all covariates (Supplementary Table 11).

No significant age-by-group interactions in CT and SD were found when comparing REs with HEs.

### Brain maturation mediates adolescent psychopathology and eating behaviors

We conducted mediation analyses to examine whether the brain differences identified above mediated the relationships between variations in IP and EP trajectories during adolescence and eating behaviors in young adulthood, as determined by the *K*-means derived clusters (the ‘psychopathology–brain maturation–eating behaviors’ models). Brain regions with significant group differences in their GMV, CT or SD trajectories were identified as regions of interest (ROIs) and tested for their mediating effects on behavioral group differences between RE or E/UEs compared with HEs. For REs, the cluster in the left cerebellum was used as the ROI. For E/UEs, differences across E/UE-related clusters were combined into a single ROI for each structural brain measure. All primary mediation analyses were adjusted for sex, recruitment sites and TIV differences to account for overall brain size variations across ages.

**Fig. 5:**
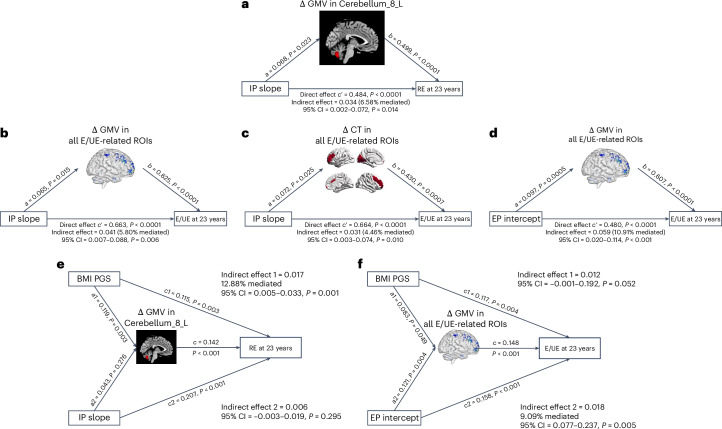
The ‘psychopathology–brain maturation–eating behaviors’ and BMI PGS–brain maturation–eating behaviors’ models. **a**, Mediation effect of GMV reductions in the left cerebellum (ages 14–23) on the relationship between age-related IP (IP slope) and RE at age 23. **b**, Mediation effects of GMV reductions on the relationship between age-related IP changes (IP slope) and E/EU at age 23, considering all E/UE-related ROIs (except the right parahippocampal gyrus, due to the different directionality of its changes compared with other ROIs). **c**, Mediation effects of CT reductions on the relationship between age-related IP changes (IP slope) and E/EU at age 23, considering all E/UE-related ROIs. **d**, Mediation effects of GMV reductions on the relationship between EP intercept and E/EU at age 23, considering all ROIs (except the right parahippocampal gyrus, due to the different directionality of its changes compared with other ROIs). **e**, The unique contribution of smaller cerebellar GMV reductions in mediating the effects of BMI PGS on restrictive eating when IP trajectory was also included in the model. **f**, The unique contribution of GMV changes in all E/UE-related ROIs (except the right parahippocampal gyrus) in the relationship between EP intercept and E/UE at age 23, beyond the effects of BMI PGS. All mediation models were adjusted for sex, recruitment sites and TIV differences (between ages 14 and 23). Cerebellum_8_L, left Lobule VIII of the cerebellar hemisphere. In the mediation models shown in panels **a**–**f**, the path coefficients *a*, *b*, *c'*, *a1*, *a2*, *c1*, *c2* and *c* represent standardized regression weights.

For REs, who differed from HEs during adolescence by their increasing trajectories of IPs (IP slope) and less pronounced GMV reductions in the left cerebellum, differences in cerebellar volume reductions partially mediated the relationship between increased IPs and being classified as an RE at age 23 (Fig. 5a). This mediation was no longer significant after adjustments for BMI, IQ and pubertal status.

Behaviorally, E/UEs differed from HEs in adolescence by their higher levels of IPs and EPs (EP and IP intercepts) and increasing trajectory of IPs (IP slope). Nominal significance was found when testing the mediation effects of GMV (Fig. 5b) and CT (Fig. 5c) differences on the relationship between IP slope and being classified as an E/UE at age 23. Mediation in the ‘IP slope–GMV–E/UE’ model remained significant after controlling for covariates, whereas for the ‘IP slope–CT–E/UE’ model, the mediation remained significant after adjusting for BMI or EA but not when adjusting for pubertal status or IQ. GMV differences in E/UE-related ROIs also significantly (*P* < 0.001) mediated the associations between EP intercept and E/UEs (Fig. 5d). This was unaffected by BMI, IQ, EA or pubertal status. No significant mediating effects were found for SD differences.

Altogether, these findings suggest that altered neurodevelopment in the RE and E/UE groups, as evidenced by protracted GMV and CT reductions, may contribute to ED symptoms, partly by mediating the effects of IPs and EPs.

### Links between genetics, brain maturation, psychopathology and eating behaviors

Considering the effects of covarying BMI in the mediation analyses above, we explored the potential contributions of polygenic risk for higher BMI. Simple ‘genetics–brain maturation–eating behaviors’ mediation models indicated that smaller GMV reductions in the RE and E/UE groups (compared with HEs) mediated the effects of BMI PGS on eating behavior profiles at age 23 in these groups (for REs versus HEs, indirect effect = 0.042, *P* = 0.002, 13.5% mediated; for E/UEs versus HEs, indirect effect = 0.043, *P* = 0.026, 11.7% mediated). For REs, this remained significant after adjusting for IQ, EA or pubertal status but not BMI. For E/UEs, this only remained significant after controlling for IQ or EA. No significant association was found between BMI PGS and CT reductions related to E/UEs. These analyses suggest that genetic predispositions to higher BMI influence BMI and restrictive and emotional/uncontrolled eating partly through their effects on protracted GMV reductions during adolescence.

Multivariate mediation analyses including IPs or EPs in these models showed the unique contribution of smaller cerebellar GMV reductions in mediating the effects of BMI PGS on REs when changes in IPs were also considered (Fig. 5e). In contrast, smaller GMV reductions in E/UEs mediated the effects of early EPs (at age 14) in this group, beyond the effects of BMI PGS (Fig. 5f). These findings suggest a specific role for cerebellar maturation in the control of BMI and restrictive eating, and additional roles for cortical and putamen maturations in mediating the effects of behavioral problems on EE and UE.

## Discussion

In this study, we used a longitudinal, multivariate analytical framework to explore the interplay among eating behaviors, genetic factors, ED-related psychopathology and brain maturation during adolescence. Our analyses revealed a high prevalence of unhealthy eating behaviors (RE and E/UE) associated with higher BMI and BMI PGS. Unhealthy eaters showed higher levels of EPs (E/UEs) and increasing trajectories of dieting (REs and E/UEs), binge eating (E/UEs) and IPs (REs and E/UEs). Age-related decreases in volumes and thickness in several brain regions, particularly in the cerebellum and prefrontal cortex (PFC), were less pronounced in REs and E/UEs compared with HEs, suggesting protracted brain maturation. Smaller volumetric reductions in the left cerebellum mediated the effects of BMI PGS on restrictive eating, even after accounting for IPs. Reductions within additional brain regions of the right hemisphere uniquely mediated the relationship between EPs and EE or UE, even after accounting for BMI PGS. These findings enhance our understanding of adolescent neurodevelopment related to ED symptomatology.

Unhealthy eaters were characterized by differing trajectories of ED symptoms during adolescence, with increasing rates of binge eating, dieting and purging in E/UEs, suggesting a greater risk for bulimia nervosa, in line with previous research^6^. Unsurprisingly^11,28^, higher levels of EPs in this group also indicated that adolescents with attention deficit hyperactivity disorder and conduct disorder symptoms may be more likely to engage in EE or UE. IPs also increased during adolescence in unhealthy eaters, notably in E/UEs, highlighting the close relationships between EE and psychological well-being^29^. Although the decreasing levels of IPs and EPs in HEs replicate previous findings^30^, differing trajectories in unhealthy eaters, already evidenced at age 14, indicate that IPs and EPs predate the development of ED symptoms.

Consistent with cortical development trajectories^31,32^, declines in structural brain measures (except for increases in SD) were observed in all eating groups, with evidence of protracted brain maturation in unhealthy eaters. Protracted brain maturation was also related to IPs and EPs, corroborating findings from children^33^, and findings of delayed trajectories of cortical thinning in children and adolescents with attention deficit hyperactivity disorder^34^. The smaller volumetric reductions in the cerebellum observed in unhealthy eaters support its role in eating pathology. Findings from anatomical, functional and behavioral studies indicate that the cerebellum is involved in the regulation of feeding behaviors and appetite control^35–38^. Disruption of a cerebellum-driven satiety network contributes to excessive eating, difficulties in stopping eating and weight gain^38^. Our finding of protracted cerebellar maturation in unhealthy eaters suggests disrupted development of such a satiety network, and engagement in restrictive eating as a compensatory mechanism to consciously control weight gain. Additional alterations in reward (right putamen) and prefrontal (for example, right PFC) circuitry might lead E/UEs to also engage in disinhibited eating due to impairments in self-regulation^39^ and impulsive action control^40^, making them more susceptible to EE or UE behaviors.

Genetic and environmental factors^41^ contribute to changes in developmental brain trajectories related to eating. Genetic risk for obesity was previously found to correlate with disordered eating and weight control behaviors in adolescents^42,43^. High BMI was also found to be associated with reduced cortical thinning in adolescence^44,45^. Our findings extend these analyses, indicating that genetic risk for obesity may influence eating-related weight control behaviors, partially via effects on cerebellar maturation, in a BMI-dependent manner.

Environmental stressors, such as adverse social environments and peer interactions, also modulate brain development^46^. Smaller GMV decreases in the cerebellum, PFC and anterior cingulate are observed in adolescents disliked by their peers, correlating with callous-unemotional traits found in externalizing disorders^47^. This altered brain development may underlie these adolescent adjustment problems and hypersensitivity to peer rejection^48^. Our findings suggest that adjustment problems influence brain maturation beyond genetic predispositions for high BMI, increasing the risk for EE and UE.

Our study’s key strengths include a well-characterized, deeply phenotyped longitudinal adolescent cohort and an innovative multivariate analytical approach. However, some limitations exist. First, the analysis is based on participants of European ancestry, necessitating future research with more ethnically diverse samples for broader applicability. Second, the use of summary scores to assess eating behaviors may oversimplify complex interactions and variations. Third, some analyses rely on VBM, which can impact GMV estimates^49^. In addition, although we considered CT and SD, we did not analyze surface area, a key volume component with distinct genetic architecture, reducing the resolution of our genetic findings.

Nonetheless, our study sheds light on how genetic risk for higher BMI, along with increasing IPs and EPs experienced during adolescence, distinctly contributes to unhealthy eating through their effects on brain maturation. The implications of these findings underscore the potential benefits of education targeting early maladaptive coping mechanisms and dietary habits to prevent EDs while promoting brain health.

## Methods

### Participants

Data analyzed in this study were collected as part of IMAGEN, a longitudinal genetic × neuroimaging cohort study of adolescents recruited from eight study centers in England, Ireland, France and Germany^50^. The IMAGEN study was approved by local research ethics committees at each study site, and written informed consent was obtained from participants and their parents or guardians. A detailed description of the study protocol and data acquisition can be found in ref. ^50^. Information on specific ethnic categories was not collected but the study, aimed at identifying the genetic and neurobiological basis of individual variability in behavior, was designed to include predominantly participants of European (white) ancestry, based on their self-reports. To further account for population stratification, statistical approaches were applied to identify and exclude genetic ancestries other than European when analyzing the genetic data. Specifically, the SDQ data used in this study were acquired at ages 14, 16, 19 and 23 years; neuroimaging data (*N* = 949) were acquired at ages 14 and 23 years and the TFEQ data were obtained at age 23 (*N* = 996).

### Neuropsychological assessments

#### Eating behaviors

The short version (18 items) of the TFEQ was used to assess eating behaviors. The TFEQ contains 3 subscales: CR, which measures the tendency to restrict one’s food intake constantly and consciously instead of using physiological cues, hunger and satiety as regulators of food intake (6 items); EE, which reflects the tendency to eat in response to negative emotions (3 items); and UE, which characterizes the tendency to overeat with the feeling of being out of control (9 items). It has good structural validity and has been used and validated in different European populations^7,51^ and was found to distinguish different eating patterns in the general population^52^.

#### ED symptoms

Dieting, binge eating and purging symptoms were assessed using the self-reports from the ED section (section P) of the Development and Well-being Assessment^21,53^. Dieting symptoms were evaluated based on responses to questions P18a, P18b and P18c, which asked about eating less at meals, skipping meals and fasting, respectively. Binge-eating symptoms were assessed using the question P15, which inquired about eating a large amount of food and losing control overeating. Purging symptoms were measured using the questions P1c, P18f and P18g, which asked about self-induced vomiting or taking pills or medicines to lose weight.

#### Emotional and behavioral problems

The SDQ was used to assess emotional and behavioral problems in adolescents. It has five hypothesized subscales, including emotional symptoms, conduct problems, hyperactivity/inattention, peer relationship problems and prosocial behaviors^54^. In low-risk and general population samples, the emotional and peer subscales can be combined into an ‘internalizing’ subscale (10 items) and the behavioral and hyperactivity subscales into an ‘externalizing’ subscale (10 items), respectively^55^. We used self-reported scores at ages 14, 16, 19 and 23 years for IPs and EPs in further analyses.

### Structural magnetic resonance imaging acquisition and processing

Magnetic resonance imaging (MRI) scans were acquired with 3T MRI scanners from different manufacturers (Siemens, Philips, General Electrics and Bruker) from 8 IMAGEN recruitment sites. The high-resolution anatomical MRI images acquired included a 3-dimensional T1-weighted magnetization prepared gradient echo sequence based on the Alzheimer’s Disease Neuroimaging Protocol (https://adni.loni.usc.edu/data-samples/adni-data/neuroimaging/mri/mri-scanner-protocols/), T2-weighted fast-spin echo and fluid-attenuated inversion recovery scans for visual assessment.

All raw images were visually inspected to exclude images with movement artifacts, brace artifacts or field inhomogeneities before preprocessing. The preprocessing procedures were then conducted using the Computational Anatomy Toolbox (CAT 12.8 (r1907); https://neuro-jena.github.io/cat/) in SPM 12 (Wellcome Department of Cognitive Neurology). We used the ‘segment longitudinal data’ procedure with default settings. Intrasubject coregistration was performed on the baseline (at age 14) and follow-up (at age 23) images. The coregistered images were then realigned across participants and bias corrected with reference to the mean images computed from each subject’s baseline and follow-up images. Next, the baseline and follow-up images and their mean images were segmented into gray matter, white matter and cerebrospinal fluid based on the default tissue classification map. Diffeomorphic Anatomical Registration Through Exponentiated Lie Algebra (DARTEL) normalization was subsequently performed on the segmented mean images using the default DARTEL template. The derived spatial normalization parameters were then applied to transform the segmented subject baseline and follow-up gray matter images into the standard Montreal Neurological Institute space. All normalized gray matter images were finally smoothed with an isotropic Gaussian kernel of 6 mm full width at half maximum. The quality measures created during preprocessing for each participant at each time point were examined, and images with sufficient quality (corresponding to grade D or above) were included in further analyses. Changes in GMV were analyzed using whole-brain VBM. Measures of CT and square root-transformed SD, which were then resampled to 12 mm in line with the recommendation for surface measures, were also derived. Longitudinal changes in mean CT and SD were extracted for different ROIs using the Desikan–Killiany atlas (*N* of ROIs = 68).

### BMI PGSs

A total of 2,087 participants were genotyped with the Illumina Human610-Quad BeadChip and Illumina Human660-Quad BeadChip during the baseline assessments. Stringent quality control (QC) procedures were performed before imputation (Supplementary Information). In brief, multidimensional scaling analysis and principal component analysis were conducted to identify genetic ancestry. Participants who were outliers from the European superpopulation were excluded (Supplementary Figs. 4 and 5) due to the limited portability across ancestries for PGSs. Consequently, 1,899 participants (49.66% male participants) who passed genotyping QC and were identified to be of European ancestry were selected for generating the BMI PGSs. IMAGEN genotype data were integrated into the European ethnicity 1KGP (phase 3 release v.5) reference panel^56^ for imputation. Summary statistics of genome-wide association study data of BMI from ~681,275 individuals of European ancestry^57^ were used to calculate BMI PGSs. This was achieved using PRS-CS^58^, which uses high-dimensional Bayesian regression and a continuous shrinkage before single-nucleotide polymorphism effect sizes. The global shrinkage parameter was set to 0.01 as is recommended for highly polygenic traits. A total of 905,362 single-nucleotide polymorphisms were used to predict BMI PGSs. Participants with available TFEQ scores were included in the subsequent analyses (for RE, *N* = 255; for E/UE, *N* = 194; for HE, *N* = 347). The BMI PGS was residualized for the first 10 principal components and batch effects before being *Z*-scored for subsequent analyses.

### Statistical analyses

#### Identification of groups with distinct eating behaviors by *K*-means clustering

*K*-means clustering using the TFEQ subscale scores (that is, CR, EE and UE) at age 23 was performed to identify clusters showing different eating behaviors. All continuous variables were transformed into *Z*-scores. We used the NbClust package to identify the optimal cluster number and validity of the cluster solution, and the fpc package to examine the clustering stability with the Jaccard coefficient and a bootstrap technique (*N* = 1000) in R.

#### Group differences in trajectories of ED symptoms across adolescence

Linear mixed models were used to examine group differences in the trajectories of dieting, binge eating and purging from ages 14 to 23. Age (that is, 14, 16, 19 and 23) was treated as a categorical variable. The models included age, group and age-by-group interactions as fixed effects and adjusted for sex. Random intercepts for participants nested within recruitment sites accounted for the dependence of repeated measures. Group-by-age interactions were investigated using HEs and age 14 as reference. A Bonferroni correction accounting for 18 tests (3 ED symptoms × 2 group comparisons × 3 age comparisons) was applied (that is, *P*_Bonferroni_ = 2.78 × 10^−^^3^).

#### Group differences in trajectories of IPs and EPs

The LGCMs were conducted in these analyses using the lavaan package in R.

##### Univariate LGCM analyses

Latent factors of intercept and slope were estimated for repeated measures (at ages 14, 16, 19 and 23 years) of IP and EP scores separately. Sex, groups and recruitment sites were considered time-invariant covariates. For these analyses, we included only those participants who had TFEQ scores at age 23 and who had at least 1 measure of IP or EP at ages 14, 16, 19 and 23. For both IP and EP, we attempted to fit a quadratic term; however, this specification resulted in a non-positive definite covariance matrix, driven by a correlation greater than or equal to one between the linear and quadratic terms. Hence, we decided not to include a quadratic term as the information contained within it was not adding any extra information over the linear term. The full information maximum likelihood estimator was used to account for data missing at random. We investigated group differences in intercepts and slopes of IP and EP trajectories, taking HEs as a reference. A Bonferroni-corrected *P*-value threshold of 0.05/(2 behaviors × 2 measures × 2 groups) = 6.25 × 10^−^^3^ was considered statistically significant.

Multivariate LGCM analyses within each group were also run to estimate models for IP and EP trajectories simultaneously and to investigate covariances between latent factors (that is, IP intercept, IP slope, EP intercept and EP slope). Sex and recruitment sites were included as covariates.

#### Longitudinal MRI analyses for group differences in brain maturation

Participants were excluded from the analysis if they had missing MRI data or failed to meet QC criteria (*N* = 47; see ‘Structural magnetic resonance imaging acquisition and processing’ for image preprocessing and QC). Consequently, a total of 949 participants (306 REs, 236 E/UEs and 407 HEs) were included in the whole-brain VBM analysis and linear mixed models for CT and SD.

##### VBM analysis

Longitudinal whole-brain VBM analyses were performed using the CAT 12.8 (r1932) toolbox. To identify brain regions reflecting significant changes in GMVs between ages 14 and 23 among the groups identified above, we performed a 2 × 2 mixed analysis of variance on the smoothed images using the ‘flexible factorial’ model. The two factors were age (age 14 or age 23; within subject) and group (that is, comparison of each of 2 groups, namely REs versus HEs, E/UEs versus HEs or REs versus E/UEs; between subject). Intracranial volumes (TIVs) were estimated by CAT 12.8 as the sum of the gray matter, white matter and cerebrospinal fluid volume. Analyses were controlled for the effects of participants’ sex, the scanning site and TIV at each time point (at ages 14 or 23). An absolute threshold masking of 0.1 was applied. The gray matter morphological differences showing significant age-by-group interactions were reported after a cluster-level, family-wise error correction with *P* < 0.05 and a cluster-forming threshold of *P* < 0.001 without correction.

##### Linear mixed models

For group differences in changes in CT and SD, we performed ROI-based linear mixed models, investigating interactions between age and groups. Models included age, groups and their interactions as fixed effects, with the participant nested within recruitment sites as a random effect and adjusted for sex. For both measures, the Bonferroni correction was applied to adjust for multiple testing (*P* = 0.05/68 ROIs × 3 group comparisons = 2.45 × 10^−4^).

#### Mediation analyses

Simple mediation models were performed using the PROCESS v.4.0 macro for R to test whether the between-group differences in brain changes mediated the relationships between differences in IP or EP trajectories and eating behaviors. We refer to this model as the psychopathology–brain maturation–eating behaviors model. Brain clusters that significantly differentiated REs from HEs, E/UEs from HEs or REs from E/UEs were considered ROIs. For group comparisons involving several brain clusters, these clusters were combined into a single ROI for each structural measure (GMV, CT or SD). For comparisons between REs and HEs, 1 mediation model was tested, relating GMV differences in the left cerebellum to differences in IP slope; therefore, a *P*-value threshold of 0.05 was considered significant. For comparisons between E/UEs and HEs, nine mediation models were tested because these two groups differed behaviorally in IP intercept, IP slope, and EP intercept, and in their changes of GMVs, CTs and SDs. The Bonferroni-corrected significance threshold of 0.05/(3 trajectory measures × 3 structural brain measures) = 5.56 × 10^−3^ was applied.

Subsequent analyses investigated the potential contributions of the BMI PGS on the brain mediation models identified above, referred to as the genetics–brain maturation–eating behaviors models. The same brain ROIs were considered as mediators in these models. For models comparing REs with HEs, the significance was set at *P* = 0.05/(1 structural measure) = 0.05. For models comparing E/UEs to HEs, the significance was set at *P* = 0.05/3 structural measures = 1.67 × 10^−2^.

Multivariate mediation models were conducted using AMOS 29 to explore the unique contributions of brain ROIs, psychopathology (IP and EP trajectories) and BMI PGS to mediation models identified in simple mediation analyses, referred to as the ‘genetics–psychopathology–brain maturation–eating behaviors’ model. Continuous variables were transformed into *Z*-scores for these analyses. CIs for the mediation effect were estimated from 5,000 bootstrap samples.

#### Covariates

Covariates for all analyses included sex and recruitment sites. For analyses involving GMV, CT and SD, TIV at the corresponding age was additionally included as a covariate. As there were no significant group differences in age at each data collection, age was considered a categorial variable in the linear mixed models and as time points in repeated measures in the LGCM analysis. For the longitudinal MRI analysis (VBM analysis and linear mixed models), participants nested within recruitment were modeled as a random effect and sex was considered a fixed effect in the model.

##### Other covariates

To examine the robustness of findings from our primary analyses, sensitivity analyses were conducted by including pubertal status, IQ, EA, and age- and sex-adjusted BMI as additional covariates. Pubertal status was assessed using the Puberty Development Scale^59^, an eight-item self-report measure of physical development based on Tanner stages, separately for male and female participants. IQ was calculated as the average of the Perceptual Reasoning Index (PRI) and Verbal Comprehension Index (VCI) scores based on age norms using the Wechsler Intelligence Scale for Children, Fourth Edition (WISC-IV; Pearson Clinical Assessment UK). We administered the block design, matrix reasoning, similarities and vocabulary subtests. Raw scores from each subtest were converted into scaled scores based on age norms. For both the PRI and VCI, we calculated prorated sums of scaled scores and then converted these sums into index scores according to the WISC-IV manual. EA was assessed by self-report of the ‘average grade at the end of the last term completed’. The age- and sex-adjusted BMI *Z*-score at age 14 was calculated using the jBmi R package based on the Centers for Disease Control and Prevention recommendations.

### Reporting summary

Further information on research design is available in the Nature Portfolio Reporting Summary linked to this article.

## Supplementary information

Supplementary Information Supplementary Information, Figs. 1–7 and Tables 1–11.

Reporting Summary

## Data Availability

Access to individual-level data from the IMAGEN project is accessible to bona fide researchers upon reasonable request and approval of a project proposal by IMAGEN consortium principal investigators. Contact the corresponding author for requests related to this study. Summary statistics from the BMI genome-wide association study, used in this study for computing BMI PGSs, are accessible via ref. ^57^ and can be downloaded from https://portals.broadinstitute.org/collaboration/giant/index.php/GIANT_consortium_data_files. Data from the 1000 Genomes Project Phase 3 may be accessed at https://www.internationalgenome.org/category/phase-3/. The Desikan–Killiany cortical atlas, used in this study for cortical parcellation, is implemented in the FreeSurfer software.

## Code availability

R (v.4.3.2) was used to perform analysis of variance and mixed linear model analysis. The lavaan R package (v.0.6-17) was used for latent growth curve modeling. Mediation analysis was conducted using the PROCESS R macro (v.4.3) and AMOS (v.20.0). MATLAB (v.2022b), SPM 12 (v.7771) and CAT12 (v.12.8 r1907) were used to process the structural MRI images and perform neuroimaging analysis. Neuroimaging results were visualized using MRIcro (v.1.9.1; https://www.nitrc.org/projects/mricro), BrainNet Viewer (v.20191031; https://www.nitrc.org/projects/bnv) and the ENIGMA visualization tool (https://github.com/MICA-MNI/ENIGMA/tree/master/enigmatoolbox). Genetic QC was performed using plink (v.1.9), the ENIGMA genotyping QC and imputation protocol (https://enigma.ini.usc.edu/wp-content/uploads/2020/02/ENIGMA-1KGP_p3v5-Cookbook_20170713.pdf), KING (v.2.3.2) and the GENESIS R package (v.2.32.0). The PGS was calculated using the publicly available PRS-cs toolbox (https://github.com/getian107/PRScs) and LD references from the 1000 Genome Project phase 3 samples with European ancestry. Other scripts used to analyze the data of this study are publicly available at https://github.com/XinyangYu918/EatingBehaviours-BrainMaturation-Psychopathology-Genetics.

## References

[1] Chan, J. K. N. et al. Life expectancy and years of potential life lost in people with mental disorders: a systematic review and meta-analysis. *eClinicalMedicine* **65**, 102294 (2023). 37965432 10.1016/j.eclinm.2023.102294 PMC10641487

[2] van Hoeken, D. & Hoek, H. W. Review of the burden of eating disorders: mortality, disability, costs, quality of life, and family burden. *Curr. Opin. Psychiatry* **33**, 521–527 (2020). 32796186 10.1097/YCO.0000000000000641 PMC7575017

[3] Piao, J. et al. Alarming changes in the global burden of mental disorders in children and adolescents from 1990 to 2019: a systematic analysis for the Global Burden of Disease study. *Eur. Child Adolesc. Psychiatry* **31**, 1827–1845 (2022). 35831670 10.1007/s00787-022-02040-4

[4] van Eeden, A. E., van Hoeken, D. & Hoek, H. W. Incidence, prevalence and mortality of anorexia nervosa and bulimia nervosa. *Curr. Opin. Psychiatry* **34**, 515–524 (2021). 34419970 10.1097/YCO.0000000000000739 PMC8500372

[5] Solmi, M. et al. Age at onset of mental disorders worldwide: large-scale meta-analysis of 192 epidemiological studies. *Mol. Psychiatry* **27**, 281 (2022). 34079068 10.1038/s41380-021-01161-7 PMC8960395

[6] Stice, E., Gau, J. M., Rohde, P. & Shaw, H. Risk factors that predict future onset of each DSM-5 eating disorder: predictive specificity in high-risk adolescent females. *J. Abnorm. Psychol.* **126**, 38–51 (2017). 27709979 10.1037/abn0000219 PMC5215960

[7] Karlsson, J., Persson, L.-O., Sjöström, L. & Sullivan, M. Psychometric properties and factor structure of the Three-Factor Eating Questionnaire (TFEQ) in obese men and women. Results from the Swedish Obese Subjects (SOS) study. *Int. J. Obes.* **24**, 1715–1725 (2000). 10.1038/sj.ijo.0801442 11126230

[8] Bryant, E. J., Rehman, J., Pepper, L. B. & Walters, E. R. Obesity and eating disturbance: the role of TFEQ restraint and disinhibition. *Curr. Obes. Rep.* **8**, 363–372 (2019). 31701348 10.1007/s13679-019-00365-x PMC6910890

[9] Cornelis, M. C. et al. Obesity susceptibility loci and uncontrolled eating, emotional eating and cognitive restraint behaviors in men and women. *Obesity* **22**, E135–E141 (2014). 23929626 10.1002/oby.20592 PMC3858422

[10] Herle, M. et al. The genomics of childhood eating behaviours. *Nat. Hum. Behav.* **5**, 625–630 (2021). 33432183 10.1038/s41562-020-01019-y PMC7610819

[11] Robinson, L. et al. Association of genetic and phenotypic assessments with onset of disordered eating behaviors and comorbid mental health problems among adolescents. *JAMA Netw. Open* **3**, e2026874 (2020). 33263759 10.1001/jamanetworkopen.2020.26874 PMC7711322

[12] Bulik, C. M. et al. Genetics and neurobiology of eating disorders. *Nat. Neurosci.* **25**, 543–554 (2022). 35524137 10.1038/s41593-022-01071-z PMC9744360

[13] Donnelly, B. et al. Neuroimaging in bulimia nervosa and binge eating disorder: a systematic review. *J. Eat. Disord.* **6**, 3 (2018). 29468065 10.1186/s40337-018-0187-1 PMC5819247

[14] Frank, G. K. W. Advances from neuroimaging studies in eating disorders. *CNS Spectr.* **20**, 391–400 (2015). 25902917 10.1017/S1092852915000012 PMC4989857

[15] King, J. A., Frank, G. K. W., Thompson, P. M. & Ehrlich, S. Structural neuroimaging of anorexia nervosa: future directions in the quest for mechanisms underlying dynamic alterations. *Biol. Psychiatry* **83**, 224–234 (2018). 28967386 10.1016/j.biopsych.2017.08.011 PMC6053269

[16] Walton, E. et al. Brain structure in acutely underweight and partially weight-restored individuals with anorexia nervosa: a coordinated analysis by the ENIGMA eating disorders working group. *Biol. Psychiatry* **92**, 730–738 (2022). 36031441 10.1016/j.biopsych.2022.04.022 PMC12145862

[17] Kessler, R. M., Hutson, P. H., Herman, B. K. & Potenza, M. N. The neurobiological basis of binge-eating disorder. *Neurosci. Biobehav. Rev.* **63**, 223–238 (2016). 26850211 10.1016/j.neubiorev.2016.01.013

[18] Berthoud, H.-R., Münzberg, H. & Morrison, C. D. Blaming the brain for obesity: integration of hedonic and homeostatic mechanisms. *Gastroenterology* **152**, 1728–1738 (2017). 28192106 10.1053/j.gastro.2016.12.050 PMC5406238

[19] Eiselt, A.-K. et al. Hunger or thirst state uncertainty is resolved by outcome evaluation in medial prefrontal cortex to guide decision-making. *Nat. Neurosci.* **24**, 907–912 (2021). 33972802 10.1038/s41593-021-00850-4 PMC8254795

[20] Hollmann, M. et al. Neural correlates of the volitional regulation of the desire for food. *Int. J. Obes.* **36**, 648–655 (2012). 10.1038/ijo.2011.125 21712804

[21] Zhang, Z. et al. Development of disordered eating behaviors and comorbid depressive symptoms in adolescence: neural and psychopathological predictors. *Biol. Psychiatry* **90**, 853–862 (2020). 32778392 10.1016/j.biopsych.2020.06.003

[22] Mitchell, K. S., Wolf, E. J., Reardon, A. F. & Miller, M. W. Association of eating disorder symptoms with internalizing and externalizing dimensions of psychopathology among men and women. *Int. J. Eat. Disord.* **47**, 860–869 (2014). 24849585 10.1002/eat.22300 PMC4237667

[23] Herpertz-Dahlmann, B. et al. Disordered eating behaviour and attitudes, associated psychopathology and health-related quality of life: results of the BELLA study. *Eur. Child Adolesc. Psychiatry* **17**, 82–91 (2008). 19132307 10.1007/s00787-008-1009-9

[24] Slane, J. D., Burt, S. A. & Klump, K. L. The road less traveled: associations between externalizing behaviors and eating pathology. *Int. J. Eat. Disord.* **43**, 149–160 (2010). 19350646 10.1002/eat.20680

[25] Allen, K. L., Byrne, S. M., Oddy, W. H. & Crosby, R. D. Early onset binge eating and purging eating disorders: course and outcome in a population-based study of adolescents. *J. Abnorm. Child Psychol.* **41**, 1083–1096 (2013). 23605960 10.1007/s10802-013-9747-7

[26] Schaumberg, K. et al. Anxiety disorder symptoms at age 10 predict eating disorder symptoms and diagnoses in adolescence. *J. Child Psychol. Psychiatry* **60**, 686–696 (2019). 30353925 10.1111/jcpp.12984 PMC6482103

[27] Cederlöf, M. et al. Etiological overlap between obsessive–compulsive disorder and anorexia nervosa: a longitudinal cohort, multigenerational family and twin study. *World Psychiatry* **14**, 333–338 (2015). 26407789 10.1002/wps.20251 PMC4592656

[28] Saif, Z. & Jahrami, H. in *Eating Disorders* (eds. Patel, V. B. & Preedy, V. R.) 123–144 (Springer International, 2023); 10.1007/978-3-031-16691-4_9

[29] Braden, A., Musher-Eizenman, D., Watford, T. & Emley, E. Eating when depressed, anxious, bored, or happy: are emotional eating types associated with unique psychological and physical health correlates? *Appetite* **125**, 410–417 (2018). 29476800 10.1016/j.appet.2018.02.022

[30] Sun, Y. et al. Associations of DNA methylation with behavioral problems, grey matter volumes and negative life events across adolescence: evidence from the longitudinal IMAGEN study. *Biol. Psychiatry* **93**, 342–351 (2022). 36241462 10.1016/j.biopsych.2022.06.012

[31] Bethlehem, Ra. I. et al. Brain charts for the human lifespan. *Nature* **604**, 525–533 (2022). 35388223 10.1038/s41586-022-04554-y PMC9021021

[32] Díaz-Caneja, C. M. et al. Sex differences in lifespan trajectories and variability of human sulcal and gyral morphology. *Cereb. Cortex* **31**, 5107–5120 (2021). 34179960 10.1093/cercor/bhab145

[33] Whittle, S., Vijayakumar, N., Simmons, J. G. & Allen, N. B. Internalizing and externalizing symptoms are associated with different trajectories of cortical development during late childhood. *J. Am. Acad. Child Adolesc. Psychiatry* **59**, 177–185 (2020). 31047992 10.1016/j.jaac.2019.04.006

[34] Shaw, P. et al. Attention-deficit/hyperactivity disorder is characterized by a delay in cortical maturation. *Proc. Natl Acad. Sci. USA* **104**, 19649–19654 (2007). 18024590 10.1073/pnas.0707741104 PMC2148343

[35] Zhu, J.-N. & Wang, J.-J. The cerebellum in feeding control: possible function and mechanism. *Cell. Mol. Neurobiol.* **28**, 469–478 (2008). 18027085 10.1007/s10571-007-9236-z PMC11515829

[36] Iosif, C. I., Bashir, Z. I., Apps, R. & Pickford, J. Cerebellar prediction and feeding behaviour. *Cerebellum* **22**, 1002–1019 (2023). 36121552 10.1007/s12311-022-01476-3 PMC10485105

[37] Sader, M., Waiter, G. D. & Williams, J. H. G. The cerebellum plays more than one role in the dysregulation of appetite: review of structural evidence from typical and eating disorder populations. *Brain Behav.* **13**, e3286 (2023). 37830247 10.1002/brb3.3286 PMC10726807

[38] Low, A. Y. T. et al. Reverse-translational identification of a cerebellar satiation network. *Nature* **600**, 269–273 (2021). 34789878 10.1038/s41586-021-04143-5 PMC8665128

[39] Heatherton, T. F. & Wagner, D. D. Cognitive neuroscience of self-regulation failure. *Trends Cogn. Sci.* **15**, 132–139 (2011). 21273114 10.1016/j.tics.2010.12.005 PMC3062191

[40] Kim, S. & Lee, D. Prefrontal cortex and impulsive decision making. *Biol. Psychiatry* **69**, 1140–1146 (2011). 20728878 10.1016/j.biopsych.2010.07.005 PMC2991430

[41] Silventoinen, K. et al. Genetic and environmental effects on body mass index from infancy to the onset of adulthood: an individual-based pooled analysis of 45 twin cohorts participating in the COllaborative project of Development of Anthropometrical measures in Twins (CODATwins) study. *Am. J. Clin. Nutr.* **104**, 371–379 (2016). 27413137 10.3945/ajcn.116.130252 PMC4962159

[42] Abdulkadir, M. et al. Polygenic score for body mass index is associated with disordered eating in a general population cohort. *J. Clin. Med.* **9**, 1187 (2020). 32326247 10.3390/jcm9041187 PMC7231239

[43] Nagata, J. M. et al. Genetic risk, body mass index, and weight control behaviors: unlocking the triad. *Int. J. Eat. Disord.* **52**, 825–833 (2019). 30994932 10.1002/eat.23083 PMC6609475

[44] Westwater, M. L., Vilar-López, R., Ziauddeen, H., Verdejo-García, A. & Fletcher, P. C. Combined effects of age and BMI are related to altered cortical thickness in adolescence and adulthood. *Dev. Cogn. Neurosci.* **40**, 100728 (2019). 31751856 10.1016/j.dcn.2019.100728 PMC6913515

[45] Kaltenhauser, S. et al. Association of body mass index and waist circumference with imaging metrics of brain integrity and functional connectivity in children aged 9 to 10 years in the US, 2016–2018. *JAMA Netw. Open* **6**, e2314193 (2023). 37200030 10.1001/jamanetworkopen.2023.14193 PMC10196880

[46] Tyborowska, A. et al. Early-life and pubertal stress differentially modulate grey matter development in human adolescents. *Sci. Rep.* **8**, 9201 (2018). 29907813 10.1038/s41598-018-27439-5 PMC6003940

[47] Blair, R. J. R., Leibenluft, E. & Pine, D. S. Conduct disorder and callous-unemotional traits in youth. *N. Engl. J. Med.* **371**, 2207–2216 (2014). 25470696 10.1056/NEJMra1315612 PMC6312699

[48] Sebastian, C., Viding, E., Williams, K. D. & Blakemore, S.-J. Social brain development and the affective consequences of ostracism in adolescence. *Brain Cogn.* **72**, 134–145 (2010). 19628323 10.1016/j.bandc.2009.06.008

[49] Antonopoulos, G. et al. A systematic comparison of VBM pipelines and their application to age prediction. *NeuroImage* **279**, 120292 (2023). 37572766 10.1016/j.neuroimage.2023.120292 PMC10529438

[50] Schumann, G. et al. The IMAGEN study: reinforcement-related behaviour in normal brain function and psychopathology. *Mol. Psychiatry* **15**, 1128–1139 (2010). 21102431 10.1038/mp.2010.4

[51] Anglé, S. et al. Three-Factor Eating Questionnaire-R18 as a measure of cognitive restraint, uncontrolled eating and emotional eating in a sample of young Finnish females. *Int. J. Behav. Nutr. Phys. Act.* **6**, 41 (2009). 19615047 10.1186/1479-5868-6-41 PMC2720907

[52] de Lauzon, B. et al. The Three-Factor Eating Questionnaire-R18 is able to distinguish among different eating patterns in a general population. *J. Nutr.* **134**, 2372–2380 (2004). 15333731 10.1093/jn/134.9.2372

[53] Goodman, R., Ford, T., Richards, H., Gatward, R. & Meltzer, H. The development and well-being assessment: description and initial validation of an integrated assessment of child and adolescent psychopathology. *J. Child Psychol. Psychiatry* **41**, 645–655 (2000). 10946756

[54] Goodman, R. The strengths and difficulties questionnaire: a research note. *J. Child Psychol. Psychiatry* **38**, 581–586 (1997). 9255702 10.1111/j.1469-7610.1997.tb01545.x

[55] Goodman, A., Lamping, D. L. & Ploubidis, G. B. When to use broader internalising and externalising subscales instead of the hypothesised five subscales on the Strengths and Difficulties Questionnaire (SDQ): data from British parents, teachers and children. *J. Abnorm. Child Psychol.* **38**, 1179–1191 (2010). 20623175 10.1007/s10802-010-9434-x

[56] 1000 Genomes Project Consortium. A global reference for human genetic variation. *Nature* **526**, 68–74 (2015). 26432245 10.1038/nature15393 PMC4750478

[57] Yengo, L. et al. Meta-analysis of genome-wide association studies for height and body mass index in ∼700,000 individuals of European ancestry. *Hum. Mol. Genet.* **27**, 3641–3649 (2018). 30124842 10.1093/hmg/ddy271 PMC6488973

[58] Ge, T., Chen, C.-Y., Ni, Y., Feng, Y.-C. A. & Smoller, J. W. Polygenic prediction via Bayesian regression and continuous shrinkage priors. *Nat. Commun.* **10**, 1776 (2019). 30992449 10.1038/s41467-019-09718-5 PMC6467998

[59] Petersen, A. C., Crockett, L., Richards, M. & Boxer, A. A self-report measure of pubertal status: reliability, validity, and initial norms. *J. Youth Adolesc.* **17**, 117–133 (1988). 24277579 10.1007/BF01537962

