# Supplementary material for: Relationships of eating behaviors with psychopathology, brain maturation and genetic risk for obesity in an adolescent cohort study
Source: Nat Ment Health. 2025 Jan 10;3(1):58–70. doi: 10.1038/s44220-024-00354-7 (PMC11726452; doi:10.1038/s44220-024-00354-7)
Supplement: Supplementary file 1 — Supplementary Information, Figs. 1–7 and Tables 1–11. [file 44220_2024_354_MOESM1_ESM.pdf]

nature mental health

Supplementary information

https://doi.org/10.1038/s44220-024-00354-7

Relationships of eating behaviors with psychopathology, brain maturation and genetic risk for obesity in an adolescent cohort study

In the format provided by the authors and unedited

Relationships between eating behaviours, psychopathology, brain maturation and genetic risk for obesity in a longitudinal adolescent cohort study

Supplementary Information

Methods **.......... 3**

Genotyping and quality control (QC) 3

Further QC and genotyping imputation 4

Results **.......... 6**

Comparisons of eating behaviours among three groups and their differences between ages 19 and 23 6

Item-level comparison of eating behaviours among three groups 6

Identification of groups with distinct eating behaviours by K-means clustering at the item 7

Supplementary figures ***11***

Supplementary Fig. S1 11

Supplementary Fig. S2 12

Supplementary Fig. S3 14

Supplementary Fig. S4 15

Supplementary Fig. S5 17

Supplementary Fig. S6 18

Supplementary Fig. S7. 20

Supplementary Tables **.......... 21**

Supplementary Table S1. Descriptive statistics of the sample for the latent growth curve model and linear mixed model analyses (available data at each data collection) 22

Supplementary Table S2. The main effects of age, group, and group-by-age interactions on disordered eating symptoms among different groups using the linear mixed model. Participants nested within each recruitment site were considered random effects, and the model controlled for sex as a fixed effect 23

Supplementary Table S3. Path estimates for IP and EP trajectories in the whole sample (Ntotal = 996; REs: N = 324; E/UEs: N = 249; HEs: N = 423). 24

Supplementary Table S4. Post-hoc analyses of IP and EP trajectories between the REs and E/UEs (Ntotal = 573; REs: N = 324; E/UEs: N = 249). 25

Supplementary Table S5. Covariances (and correlations) among the IP and EP intercepts and slopes in each group. 26

Supplementary Table S6. Differences in GMV development between groups. 27

Supplementary Table S7. The age-by-group interactions of GMV changes among different groups using the mixed linear models. RE, restrictive eaters; E/UE, emotional and uncontrolled eaters; HE,

healthy eaters. The primary analysis was adjusted for sex, recruitment sites, total intracranial volume......28

Supplementary Table S8. The age-by-group interactions of CT changes among different groups using the mixed linear models. RE, restrictive eaters; E/UE, emotional and uncontrolled eaters; HE, healthy eaters. The analyses were adjusted for sex, recruitment sites, total intracranial volume. 30

Supplementary Table S9. The age-by-group interactions of CT changes among different groups using the mixed linear models, accounting for the effects of pubertal status, IQ, educational attainment, and age- and sex-adjusted BMI Z-score, and Euler's numbers. RE, restrictive eaters; E/UE, emotional and uncontrolled eaters; HE, healthy eaters. [a]The column "remain sig.' indicates whether the primary analyses remain Bonferroni significant (p < 0.05/(68*3) = 2.45×10-4) after adjusting for the corresponding covariates. 34

Supplementary Table S10. The age-by-group interactions of SD changes among different groups using the mixed linear models. RE, restrictive eaters; E/UE, emotional and uncontrolled eaters; HE, healthy eaters. The analyses were adjusted for sex, recruitment sites, total intracranial volume. 37

Supplementary Table S11. The age-by-group interactions of SD changes among different groups using the mixed linear models, accounting for the effects of pubertal status, IQ, educational attainment, and age- and sex-adjusted BMI Z-score, and Euler's numbers. RE, restrictive eaters; E/UE, emotional and uncontrolled eaters; HE, healthy eaters. [a]The column "remain sig.' indicates whether the primary analyses remain Bonferroni significant ($p < 0.05/(68*3) = 2.45\times10^{-4}$) after adjusting for the corresponding covariates. 41

***References* 44**

Methods

Genotyping and quality control (QC)

DNA purification and genotyping were carried out by the Centre National de Génotypage in Paris. DNA was extracted from whole-blood samples (~10 ml) preserved in BD Vacutainer EDTA tubes (Becton, Diskinson and Company) using the Gentra Puregene Blood Kit (QIAGEN), following the manufacturer's instructions. A total of 705 and 1382 individuals were genotyped with the Illumina (Little Chesterford, UK) Human610-Quad BeadChip and Illumina Human660-Quad BeadChip, respectively. Genotype information was collected at 582,982 markers. For each genotyping platform, initial quality control was performed separately. Single-nucleotide polymorphisms (SNPs) with call rates < 95%, minor allele frequency < 5%, deviation from the Hardy–Weinberg equilibrium ($P \leqslant 1 \times 10^{-3}$) and non-autosomal SNPs were excluded from the analyses. Individuals with excessive missing genotypes (failure rate > 5%) were also excluded. Population homogeneity was examined with the Structure software using HapMap populations as reference groups[1]. Individuals with divergent ancestry (from Utah residents with ancestry from northern and western Europe) were excluded. Identity-by-state clustering and multi-dimensional scaling were used to estimate cryptic relatedness for each pair of individuals using the PLINK software[2] and closely related individuals were eliminated from the subsequent analysis. Principal component analysis was applied to remove remaining outliers[3], defined as individuals located at more than four standard deviations of the mean principal component analysis scores on one of the first 20 dimensions. The genotypes from both Illumina Human610 Quad BeadChip and Human660-Quad BeadChip were combined, and platform-specific SNPs were removed. Standard QC procedures following the ENIGMA protocol[4] were again conducted on the merged data. This included removing SNPs with Minor Allele Frequency < 0.01, Genotype Call Rate < 95%, Hardy-Weinberg Equilibrium < 1×10^{-6} and strand ambiguous and duplicated SNPs. No individuals had a

reported sex that did not match their genetic sex. Participants exhibiting excessive heterozygosity (± 3 SD from the mean; $N = 26$) were excluded. IMAGEN excluded by design siblings or any related individuals; however, when known sibling relationships were identified, those individuals were also excluded. Genetic homogeneity was assessed using multi-dimensional scaling (MDS) analysis, which involved merging data from the HapMap 3 populations with the IMAGEN dataset, and then calculating MDS components from the combined data set (**Supplementary Fig. S4**). Ancestry outliers ($N = 143$) were identified and excluded based on a visual inspection of the first two MDS components[5].

Further QC and genotyping imputation

IMAGEN genotype data was integrated into the European ethnicity 1KGP (phase 3 release v5) reference panel[6] for imputation. Prior to imputation, the genetic relatedness and population structure of the data were further assessed by computing a kinship relatedness matrix for each pair using the KING software[7]. The GENESIS package[8] was used to perform principal component (PC) analysis, accounting for relatedness in the sample, and identify PCs that accurately capture population structure but not family structure. The PCA plot was used to visualise clusters of participants from different genetic ancestries based on their PCs (**Supplementary Fig. S5**). Fifteen outliers from the European ancestry among the IMAGEN participants were excluded from subsequent analyses. Genetic relatedness of participants who passed the QC was estimated again, and genetic PCs were recalculated after accounting for relatedness.

A total of 1899 participants (943 males and 956 female participants) who passed the QC were selected for genotyping imputation. Imputation against the 1KGP (phase 3 release v5) reference panel was performed using the Michigan Imputation Server (Minimac4)[9] and Eagle v2.4 for

phasing, targeting the European population. Following imputation, SNPs with poor imputation quality (info < 0.4), or with a minor allele frequency < 0.1% were removed from the imputed data. The remaining data were finally combined into a single file for polygenic scoring.

Results

Comparisons of eating behaviours among three groups and their differences between ages 19 and 23

Eating behaviours (cognitive restraints, CR; emotional eating, EE; and uncontrolled eating, UE) were accessed at ages 19 and 23, not at earlier ages. To test whether eating behaviours changed over time in the defined groups, we compared eating behaviours in these groups at ages 19 and 23y, using paired t-tests.

Between-group comparisons indicated that the three groups had consistent eating profiles across time. Both at ages 19 and 23y, REs exhibited the highest CR, E/UEs had an intermediate level, and HEs showed the lowest CR. E/UEs had the highest levels of EE and UE compared to the other two groups. REs displayed more EE behaviours (but not UE) than HEs (all ps < 0.001) (**Supplementary Fig. S2**). Within groups comparisons indicated that in contrast to HEs that reported decreases in all three eating behaviours from ages 19 to 23 (CR: Cohen's d = -0.43, $p = 6.43 \times 10^{-12}$; EE: Cohen's d = -0.43, $p = 8.93 \times 10^{-13}$: UE: Cohen's d = -0.34, $p = 1.28 \times 10^{-16}$), unhealthy eating increased in the other two groups: CR increased in REs (Cohen's d = 0.38, $p = 4.07 \times 10^{-14}$), while EE and UE increased in E/UEs (EE: Cohen's d = 0.46, $p = 2.56 \times 10^{-13}$; UE: Cohen's d = 0.36, $p = 4.42 \times 10^{-9}$).

Item-level comparison of eating behaviours among three groups

We assessed differences in eating behaviours using all 18 items from the TFEQ-R18 across different groups (see list of TFEQ-R18 items below). The TFEQ-R18 includes 6 items measuring CR, 3 for EE, and 9 for UE. We performed logistic regressions with the group as a dependent variable, and calculated the odds ratios (ORs) and 95% confidence intervals for each

item between each pair of groups, and applied the Bonferroni correction for multiple comparisons ($p_{\text{Bonferroni}} = 0.05/(3 \text{ groups} \times 18 \text{ items}) = 9.26 \times 10^{-4}$; **Supplementary Fig. S6**).

REs vs. E/UEs: REs had significantly higher ORs in all CR-related items and significantly lower ORs in all EE- and UE-related items, when compared to E/UEs.

REs vs. HEs: When compared to HEs, REs had significantly higher ORs at all CR-related items. They also had higher, albeit less significant, ORs in all EE-related items, and four of the UE-related items (items 4, 8, 9, and 17). The remaining UE-related items showed nominally higher ORs ($p < 0.05$). Behaviours that differentiated REs most from HEs (ORs > 5) included consciously eating less to control weight or not gain weight, the intensity of restraint in eating, consciously eating less than wanted, and not eating foods that made them fat.

E/UEs vs. HEs: Compared to HEs, E/UEs displayed significantly higher ORs in all EE-related items, followed by all UE-related items, and in most CR-related items, except for item 18, which reached nominal significance with a p-value of 2.79×10^{-3}. Behaviours that differentiated E/UEs most from HEs (ORs > 5) included eating/overeating when feeling blue, lonely or anxious (all EE items), inability to stop eating, and frequency of binge eating episodes (UE items).

Identification of groups with distinct eating behaviours by K-means clustering at the item level

To address the complexities and potential limitations associated with the use of sum scores[10] in our study, We repeated our K-means clustering analysis based on all 18 items from the TFEQ individually. Again, using the "NbClust" package, we identified three clusters as the optimal number of classes, determined by multiple indices. Consistent with our findings based on sum scores, three distinct groups with different eating behaviour profiles emerged: one cluster (previously defined as restrictive eaters) exhibited the highest scores on all CR-related items,

a second cluster (defined as emotional and uncontrolled eaters) showed the highest scores on all EE and UE-related items, and the third cluster (defined as healthy eaters) scored low on all items (**Supplementary Fig. S7**).

The sample sizes for each group identified by individual TFEQ items matched those identified by TFEQ sum scores, as presented in the Table below. On average, 81.02% of the participants (807 out of 996) were consistently classified into the same group whether using sum scores or item scores across the three groups.

Group	RE group	E/UE group	HE group
Definition	Participants scored high in CR-related items or CR subscale, compared to the other two groups	Participants scored high in EE and UE-related items or EE and UE subscales, compared to the other two groups	Participants scored low in all TFEQ items or all subscales
N, identified by sum scores	324	249	423
N, identified by item scores	306	283	407
N (%) of participants whose group identification by sum scores matched their identification by item scores	257 (79.32%)	194 (77.91%)	356 (84.16%)

<u>List of items from the TFEQ Revised 18 questionnaire:</u>

1. When I smell a delicious food, I find it very difficult to keep from eating, even if I have just finished a meal.

    Definitely true (4)/ mostly true (3)/ mostly false (2)/ definitely false (1)

2. I deliberately take small helpings as a means of controlling my weight.

    Definitely true (4)/ mostly true (3)/ mostly false (2)/ definitely false (1)

3. When I feel anxious, I find myself eating.

    Definitely true (4)/ mostly true (3)/ mostly false (2)/ definitely false (1)

4. Sometimes when I start eating, I just can’t seem to stop.

    Definitely true (4)/ mostly true (3)/ mostly false (2)/ definitely false (1)

5. Being with someone who is eating often makes me hungry enough to eat also.

Definitely true (4)/ mostly true (3)/ mostly false (2)/ definitely false (1)

6. When I feel blue, I often overeat.

Definitely true (4)/ mostly true (3)/ mostly false (2)/ definitely false (1)

7. When I see a real delicacy, I often get so hungry that I have to eat right away.

Definitely true (4)/ mostly true (3)/ mostly false (2)/ definitely false (1)

8. I get so hungry that my stomach often seems like a bottomless pit.

Definitely true (4)/ mostly true (3)/ mostly false (2)/ definitely false (1)

9. I am always hungry so it is hard for me to stop eating before I finish the food on my plate.

Definitely true (4)/ mostly true (3)/ mostly false (2)/ definitely false (1)

10. When I feel lonely, I console myself by eating.

Definitely true (4)/ mostly true (3)/ mostly false (2)/ definitely false (1)

11. I consciously hold back at meals in order not to weight gain.

Definitely true (4)/ mostly true (3)/ mostly false (2)/ definitely false (1)

12. I do not eat some foods because they make me fat.

Definitely true (4)/ mostly true (3)/ mostly false (2)/ definitely false (1)

13. I am always hungry enough to eat at any time.

Definitely true (4)/ mostly true (3)/ mostly false (2)/ definitely false (1)

14. How often do you feel hungry?

Only at meal times (1)/ sometimes between meals (2)/ often between meals (3)/almost always (4)

15. How frequently do you avoid “stocking up” on tempting foods?

Almost never (1)/ seldom (2)/ moderately likely (3)/ almost always (4)

16. How likely are you to consciously eat less than you want?

Unlikely (1)/ slightly likely (2)/ moderately likely (3)/ very likely (4)

17. Do you go on eating binges though you are not hungry?

Never (1)/ rarely (2)/ sometimes (3)/ at least once a week (4)

18. On a scale of 1 to 8, where 1 means no restraint in eating (eating whatever you want, whenever you want it) and 8 means total restraint (constantly limiting food intake and never "giving in"), what number would you give yourself?

Supplementary figures

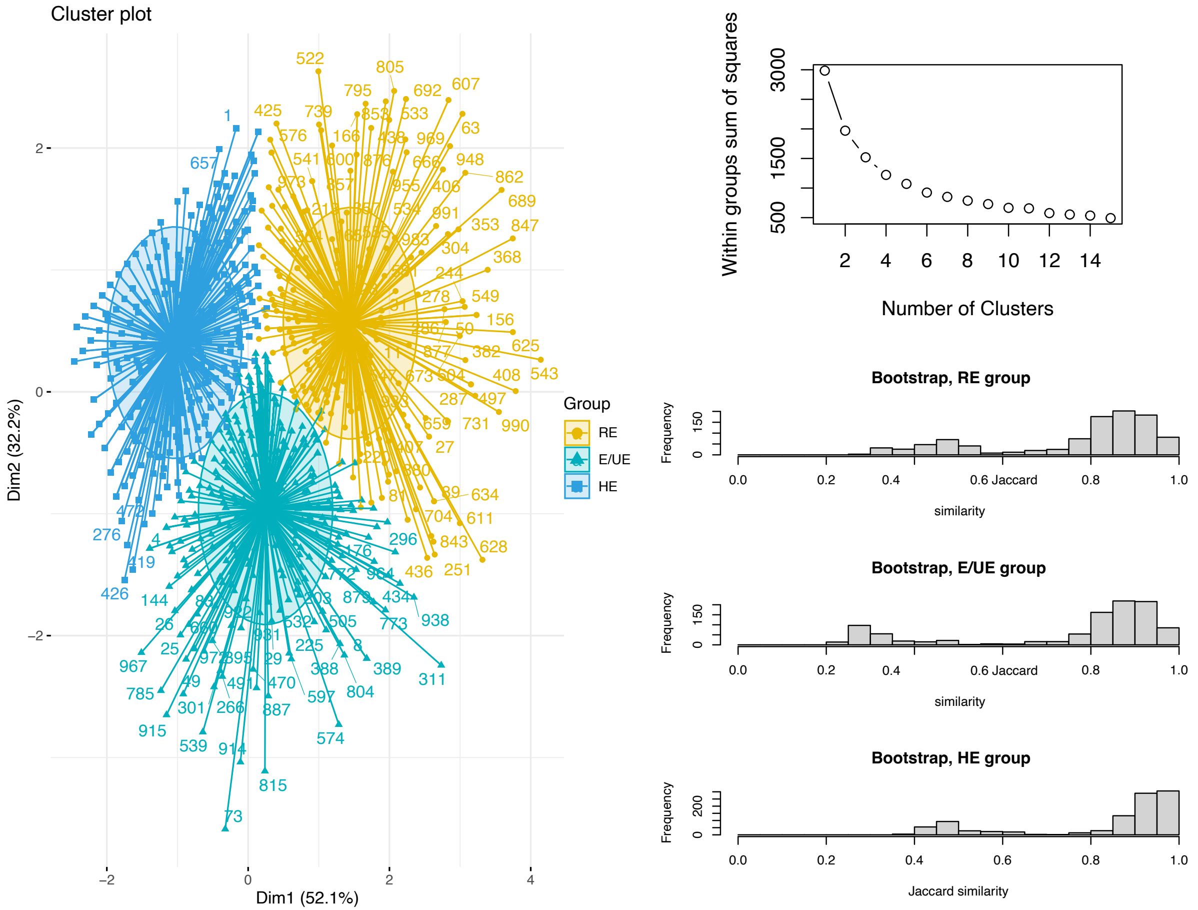

Supplementary Fig. S1

Cluster distribution and Jaccard similarity (N = 1000 bootstraps) of the three eating groups identified from K-means clustering analysis.

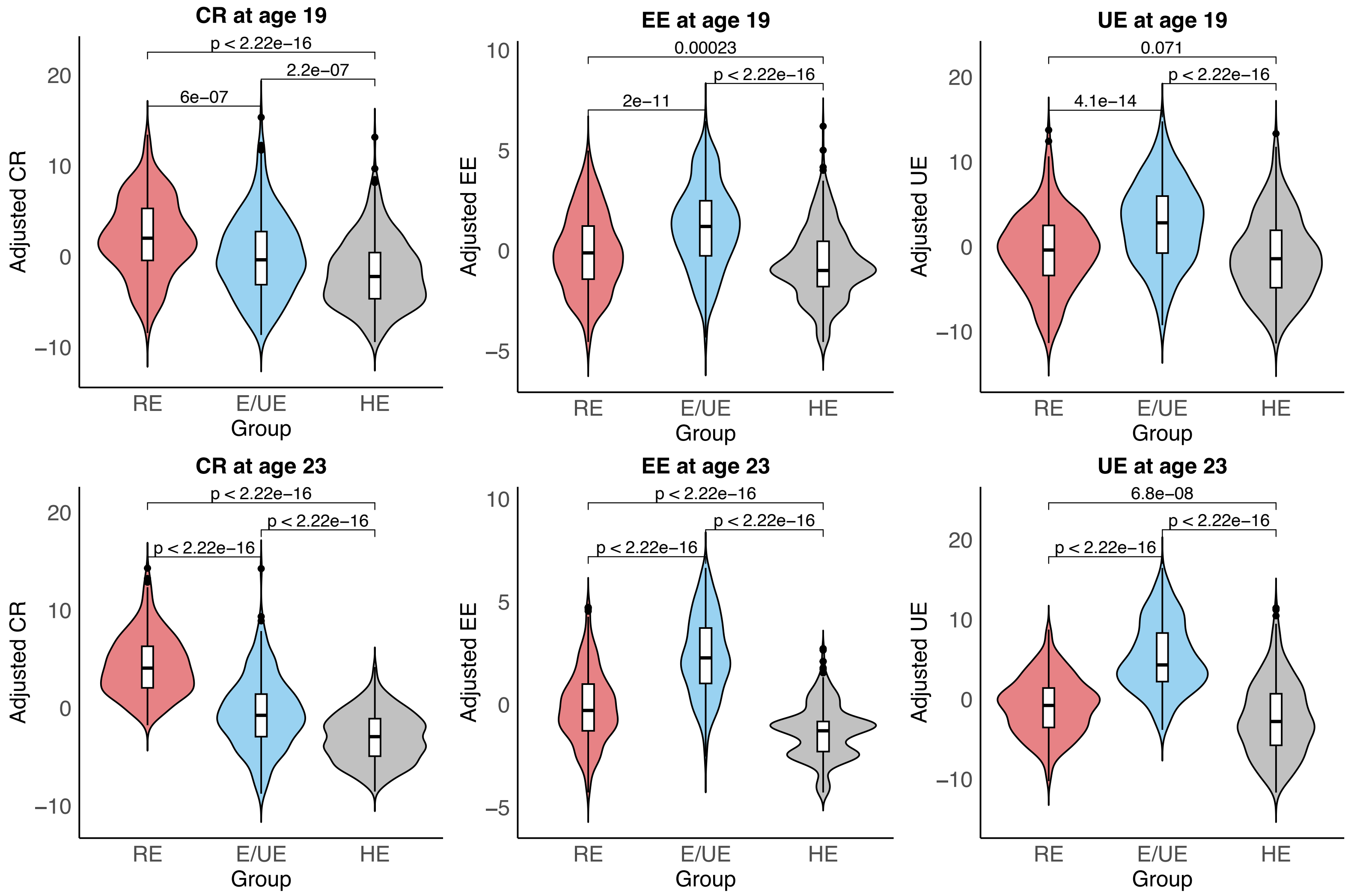

Supplementary Fig. S2

Group differences in eating behaviours (cognitive restraints, CR; emotional eating, EE; uncontrolled eating, UE) at ages 19 and 23.

The sample sizes for restrictive eaters (RE), emotional and uncontrolled eaters (E/UE) and healthy eaters (HE) at age 19 are 279, 215, and 362, respectively; at age 23, the sample sizes are 324, 249, and 423, respectively. Group comparisons (analysis of variance, ANOVA; two-sided) were adjusted for age, sex, and recruitment sites. Multiple comparisons were conducted using Bonferroni correction. The box plots display the minima (lower whisker), maxima (upper whisker), median (centre line), and outliers (points beyond the whiskers) for each group.

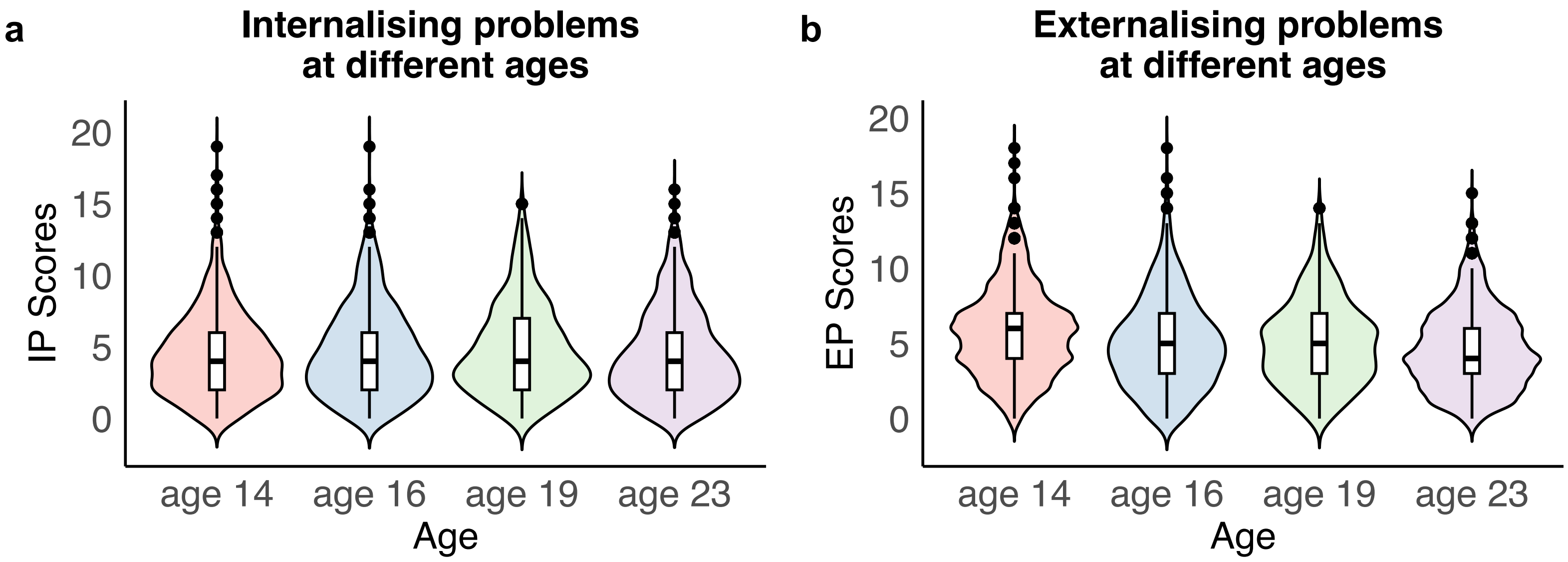

Supplementary Fig. S3

Descriptive plots of internalising problem (IP; **a**) and externalising problem (EP; **b**) scores at different ages in the whole sample. The sample sizes for internalising and externalising at ages 14, 16, 19 and 23 are 995, 888, 900, and 982, respectively. The box plots display the minima (lower whisker), maxima (upper whisker), median (centre line), and outliers (points beyond the whiskers) for each age.

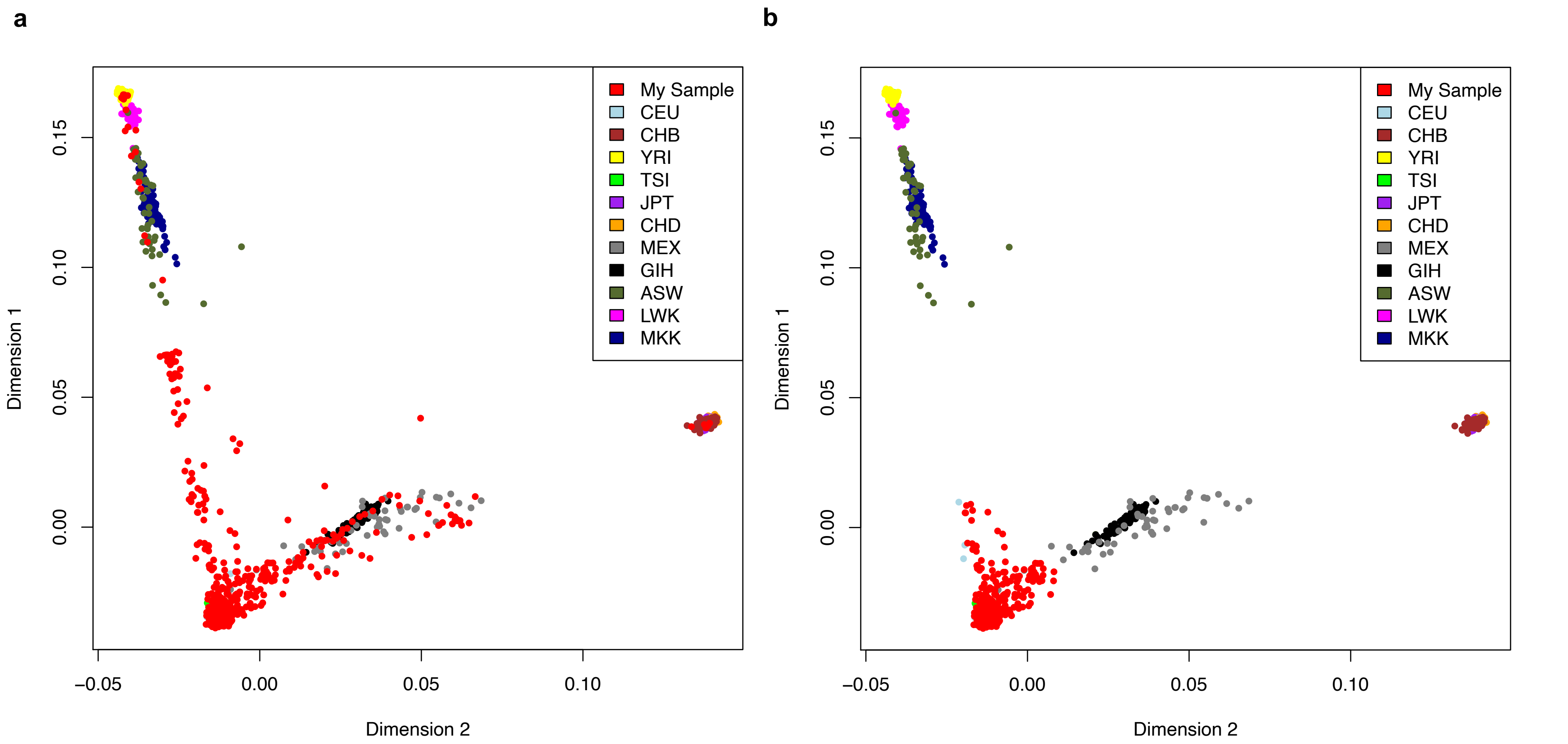

Supplementary Fig. S4

Ancestry inference via multi-dimensional scaling plots in the whole sample. MDS plots of the IMAGEN participants to HapMap III reference panels of known ancestry are displayed. ASW, African ancestry in southwest USA; CEU, Utah residents with northern and western European

ancestry from the CEPH collection; CHD, Chinese in metropolitan Denver, Colorado; GIH, Gujarati Indians in Houston, Texas; LWK, Luhya in Webuye, Kenya; MEX, Mexican ancestry in Los Angeles, California; MKK, Maasai in Kinyawa, Kenya; TSI, Tuscans in Italy; YRI, Yoruba in Ibadan, Nigeria. **a**, The original MDS plot for all IMAGEN participants. **b**, The MDS plot after removal of the 143 participants not clustering together with the CEU participants.

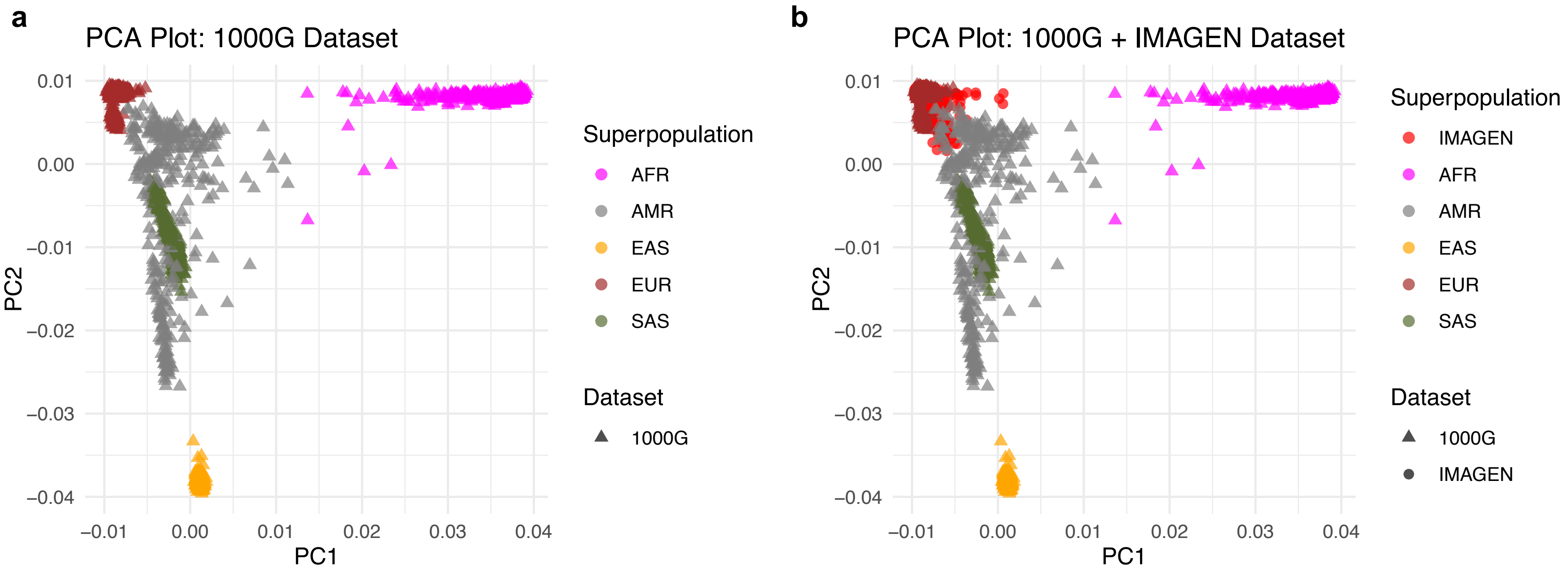

Supplementary Fig. S5

Assessment of genetic homogeneity in the IMAGEN cohort. The IMAGEN dataset was merged with the 1000 Genomes Project Phase 3 dataset, and a kinship relatedness matrix was calculated for each pair using the KING software. To account for relatedness within the sample, principal component (PC) analysis was conducted using the GENESIS R package. PCA plot was used to visualise clusters of participants from different genetic ancestries based on their PCs. AFR, African; AMR, Admixed American; EAS, East Asian; EUR, European; SAS, South Asian. **a**, PCA plot of 1000 Genomes participants. **b**, PCA plot of the combined samples of 1000 Genome participants and IMAGEN participants after excluding participants identified as outliers from the EUR superpopulation.

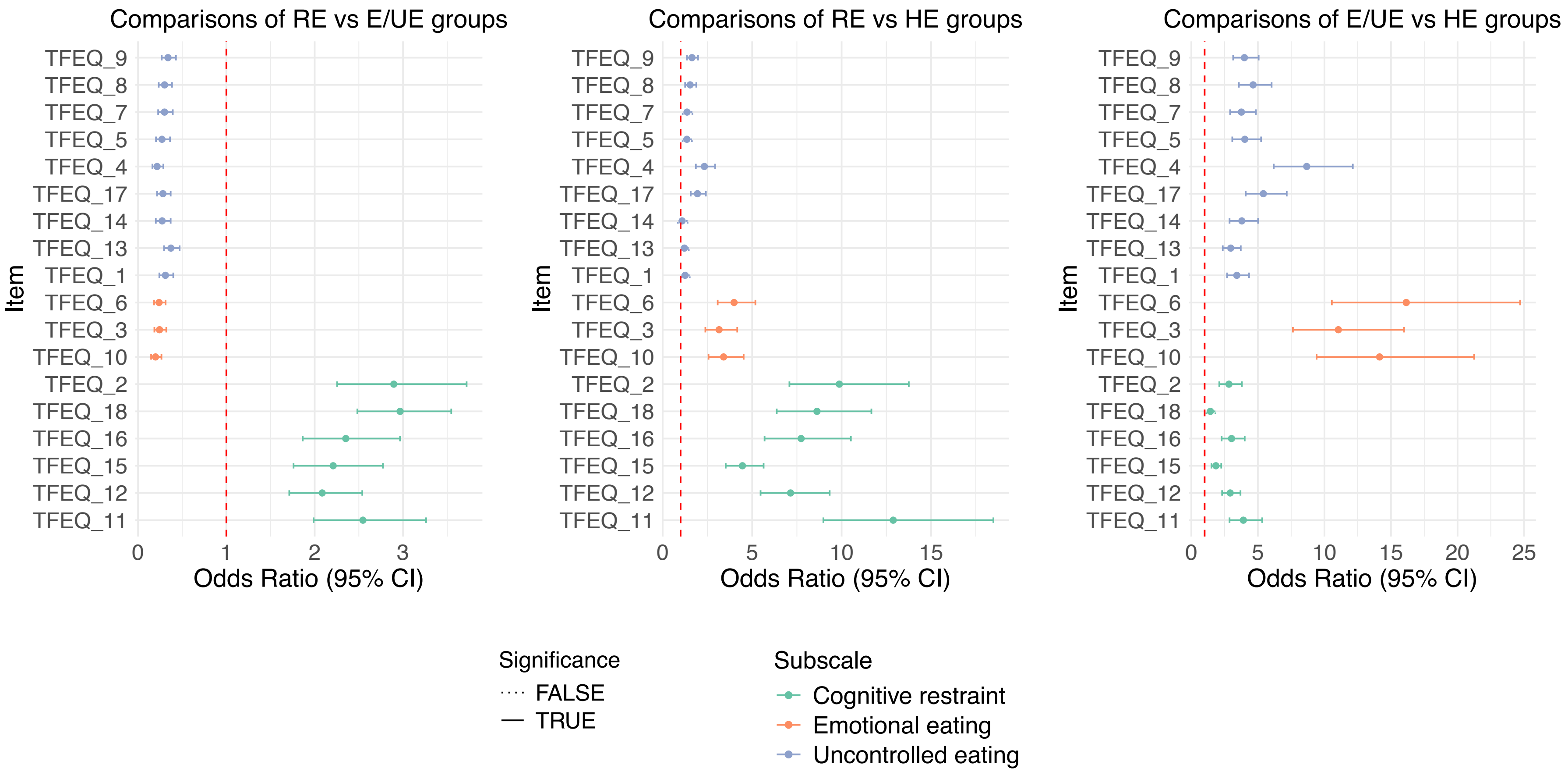

Supplementary Fig. S6

The figure displays the odd ratios (ORs) and 95% confidence intervals (CIs) for different Three Factor Eating Questionnaire (TFEQ) items across three group comparisons: restrictive eaters (RE) vs emotional and uncontrolled eaters (E/UE; left panel), restrictive eaters (RE) vs healthy eaters (HE; middle panel), emotional and uncontrolled eaters (E/UE) vs healthy eaters (HE; right panel). Data are presented as mean ORs ± 95% CIs.

The sample sizes for RE, EE, and HE are 324, 249 and 423, respectively. Solid lines represent significant group comparisons and dashed lines indicate non-significant comparisons.

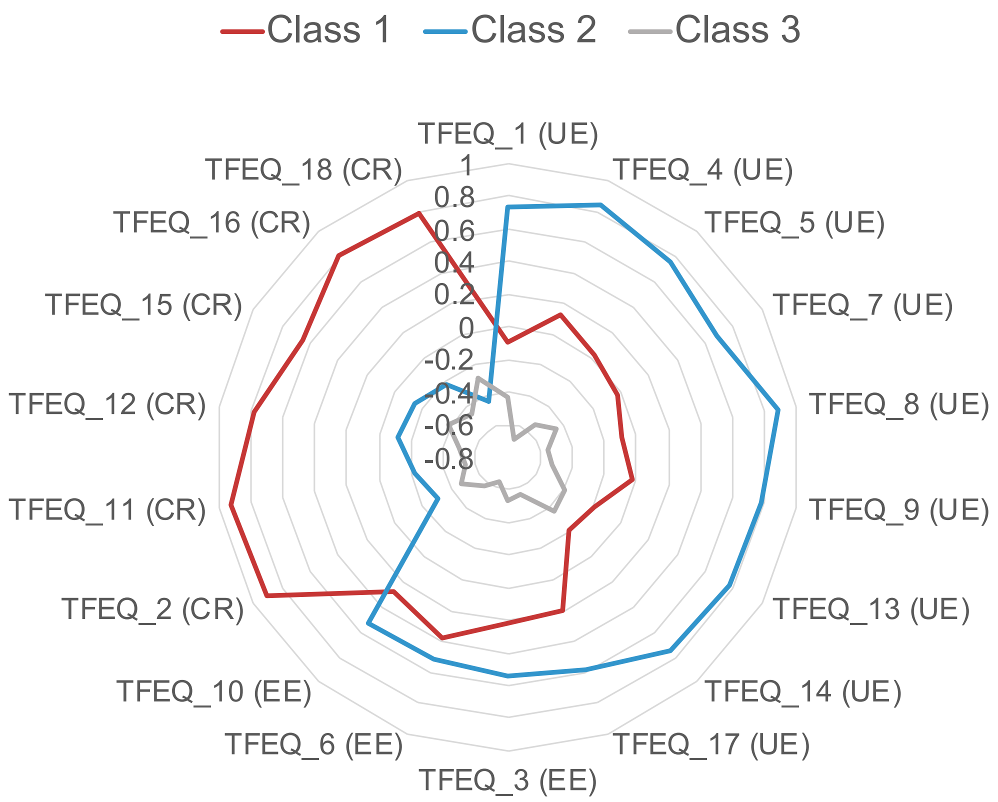

Supplementary Fig. S7.

Distinct groups of different eating behaviours identified by K-means clustering analysis based on all 18 items from the TFEQ questionnaire. Three clusters were identified, consistent with the clustering results based on the three 3 subscale sum scores. CR, cognitive restraint; EE, emotional eating; UE, uncontrolled eating.

Supplementary Tables

Contents	
**Supplementary Table S1**	Descriptive statistics of the sample for the latent growth curve model and linear mixed model analyses (available data at each data collection).
**Supplementary Table S2**	The main effects of age, group, and group-by-age interactions on disordered eating symptoms among different groups using the linear mixed model. Participants nested within each recruitment site were considered random effects, and the model controlled for sex as a fixed effect.
**Supplementary Table S3**	Path estimates for IP and EP trajectories in the whole sample (Ntotal = 996; REs: N = 324; E/UEs: N = 249; HEs: N = 423).
**Supplementary Table S4**	Post-hoc analyses of IP and EP trajectories between the REs and E/UEs (Ntotal = 573; REs: N = 324; E/UEs: N = 249).
**Supplementary Table S5**	Covariances (and correlations) among the IP and EP intercepts and slopes in each group.
**Supplementary Table S6**	Differences in GMV development between groups.
**Supplementary Table S7**	The age-by-group interactions of GMV changes among different groups using the mixed linear models. RE, restrictive eaters; E/UE, emotional and uncontrolled eaters; HE, healthy eaters. The primary analysis was adjusted for sex, recruitment sites, total intracranial volume.
**Supplementary Table S8**	The age-by-group interactions of CT changes among different groups using the mixed linear models. RE, restrictive eaters; E/UE, emotional and uncontrolled eaters; HE, healthy eaters. The analyses were adjusted for sex, recruitment sites, total intracranial volume.
**Supplementary Table S9**	The age-by-group interactions of CT changes among different groups using the mixed linear models, accounting for the effects of pubertal status, IQ, educational attainment, and age- and sex-adjusted BMI Z-score, and Euler's numbers. RE, restrictive eaters; E/UE, emotional and uncontrolled eaters; HE, healthy eaters. [a]The column "remain sig.' indicates whether the primary analyses remain Bonferroni significant ($p < 0.05/(68*3) = 2.45\times10^{-4}$) after adjusting for the corresponding covariates.
**Supplementary Table S10**	The age-by-group interactions of SD changes among different groups using the mixed linear models. RE, restrictive eaters; E/UE, emotional and uncontrolled eaters; HE, healthy eaters. The analyses were adjusted for sex, recruitment sites, total intracranial volume.
**Supplementary Table S11**	The age-by-group interactions of SD changes among different groups using the mixed linear models, accounting for the effects of pubertal status, IQ, educational attainment, and age- and sex-adjusted BMI Z-score, and Euler's numbers. RE, restrictive eaters; E/UE, emotional and uncontrolled eaters; HE, healthy eaters. [a]The column "remain sig.' indicates whether the primary analyses remain Bonferroni significant ($p < 0.05/(68*3) = 2.45\times10^{-4}$) after adjusting for the corresponding covariates.

Supplementary Table S1. Descriptive statistics of the sample for the latent growth curve model and linear mixed model analyses (available data at each data collection).

	N (% of the whole sample)	Mean	SD
Baseline (age 14)			
Internalising problems	995 (99.90%)	4.43	2.95
Externalising problems	995 (99.90%)	5.77	2.95
Dieting symptoms	996 (100%)	0.06	0.23
Binge eating symptoms	996 (100%)	0.03	0.18
Purging symptoms	996 (100%)	0.03	0.17
Follow-up 1 (age 16)			
Internalising problems	888 (89.16%)	4.62	3.13
Externalising problems	888 (89.16%)	4.99	3.02
Dieting symptoms	888 (89.16%)	0.09	0.28
Binge eating symptoms	888 (89.16%)	0.07	0.26
Purging symptoms	888 (89.16%)	0.06	0.25
Follow-up 2 (age 19)			
Internalising problems	900 (90.36%)	4.78	3.15
Externalising problems	900 (90.36%)	5.06	2.82
Dieting symptoms	900 (90.36%)	0.08	0.27
Binge eating symptoms	900 (90.36%)	0.07	0.26
Purging symptoms	900 (90.36%)	0.05	0.21
Follow-up 3 (age 23)			
Internalising problems	982 (98.59%)	4.47	3.12
Externalising problems	982 (98.59%)	4.60	2.66
Dieting symptoms	982 (98.59%)	0.04	0.20
Binge eating symptoms	982 (98.59%)	0.07	0.25
Purging symptoms	982 (98.59%)	0.04	0.20

Supplementary Table S2. The main effects of age, group, and group-by-age interactions on disordered eating symptoms among different groups using the linear mixed model. Participants nested within each recruitment site were considered random effects, and the model controlled for sex as a fixed effect.

	beta	se	*t*	*p*	$p_{Bonferroni}$
Dieting symptoms					
age16	-0.004	0.016	-0.243	0.808	
age19	-0.007	0.016	-0.446	0.656	
age23	-0.028	0.015	-1.839	0.066	
RE	0.049	0.018	2.734	0.006	
E/UE	0.012	0.020	0.605	0.545	
age16:RE	0.043	0.024	1.753	0.080	1.000
age19:RE	0.019	0.024	0.788	0.431	1.000
age23:RE	0.001	0.024	0.035	0.972	1.000
age16:E/UE	0.083	0.026	3.184	0.001	0.026
age19:E/UE	0.070	0.026	2.680	0.007	0.133
age23:E/UE	0.030	0.025	1.174	0.240	1.000
Binge eating symptoms					
age16	0.023	0.015	1.536	0.125	
age19	0.006	0.015	0.381	0.704	
age23	-0.007	0.014	-0.501	0.617	
RE	0.024	0.017	1.355	0.175	
E/UE	0.031	0.019	1.648	0.099	
age16:RE	0.019	0.023	0.830	0.406	1.000
age19:RE	0.041	0.022	1.816	0.069	1.000
age23:RE	0.025	0.022	1.154	0.249	1.000
age16:E/UE	0.060	0.024	2.458	0.014	0.253
age19:E/UE	0.077	0.024	3.169	0.002	0.028
age23:E/UE	0.134	0.024	5.659	1.68E-08	3.024E-07
Purging symptoms					
age16	0.014	0.013	1.106	0.269	
age19	3.85E-04	0.013	0.030	0.976	
age23	-0.005	0.012	-0.384	0.701	
RE	0.022	0.015	1.465	0.143	
E/UE	0.020	0.016	1.251	0.211	
age16:RE	0.018	0.020	0.940	0.347	1.000
age19:RE	0.017	0.019	0.878	0.380	1.000
age23:RE	0.012	0.019	0.658	0.510	1.000
age16:E/UE	0.062	0.021	2.959	0.003	0.056
age19:E/UE	0.046	0.021	2.207	0.027	1.000
age23:E/UE	0.042	0.020	2.034	0.042	1.000

Supplementary Table S3. Path estimates for IP and EP trajectories in the whole sample (Ntotal = 996; REs: N = 324; E/UEs: N = 249; HEs: N = 423).

	Estimate	SE	95% confidence intervals		*p* value
IP model					
IP intercept					
RE	0.253	0.199	-0.137	0.642	0.203
E/UE	0.712	0.220	0.281	1.144	0.001
IP slope					
RE	0.073	0.027	0.019	0.126	0.008
E/UE	0.110	0.030	0.051	0.169	< 0.001
EP model					
EP intercept					
RE	0.092	0.202	-0.303	0.487	0.648
E/UE	0.855	0.223	0.418	1.292	< 0.001
EP slope					
RE	0.011	0.024	-0.037	0.059	0.650
E/UE	0.035	0.027	-0.017	0.088	0.189

For the IP model, $\chi2(25) = 81.723$; CFI = 0.955; TLI = 0.918; RMSEA = 0.048; SRMR = 0.021. For the EP model, $\chi2(25) = 90.115$; CFI = 0.948; TLI = 0.908; RMSEA = 0.051; SRMR = 0.020. IP, internalising problems; EP, externalising problems. RE and E/UE were two dummy-coded variables with the HE group as reference. The model was controlled for sex and recruitment sites as time-invariant covariates.

Supplementary Table S4. Post-hoc analyses of IP and EP trajectories between the REs and E/UEs (Ntotal = 573; REs: N = 324; E/UEs: N = 249).

	Estimate	SE	95% confidence intervals		*p* value
IP model					
IP intercept					
E/UE	0.444	0.236	-0.019	0.906	0.060
IP slope					
E/UE	0.038	0.031	-0.024	0.099	0.228
EP model					
EP intercept					
E/UE	0.743	0.237	0.278	1.209	0.002
EP slope					
E/UE	0.031	0.029	-0.026	0.088	0.290

For the IP model, $\chi2(23) = 49.622$; CFI = 0.962; TLI = 0.931; RMSEA = 0.045; SRMR = 0.022. For the EP model, $\chi2(23) = 57.755$; CFI = 0.950; TLI = 0.908; RMSEA = 0.051; SRMR = 0.021. IP, internalising problems; EP, externalising problems. E/UE was a dummy-coded variable with the RE group as reference. The model was controlled for sex and recruitment sites as time-invariant covariates.

Supplementary Table S5. Covariances (and correlations) among the IP and EP intercepts and slopes in each group.

	Estimate (covariances)	SE	95% confidence intervals		*p* value	Standarised covariances (i.e., correlation *r*)
In the RE group						
IP intercept						
IP slope	-0.234	0.069	-0.369	-0.098	0.001	-0.522
EP intercept	1.479	0.440	0.617	2.341	0.001	0.295
EP slope	-0.071	0.055	-0.179	0.037	0.195	-0.158
IP slope						
EP intercept	-0.101	0.061	-0.220	0.019	0.098	-0.203
EP slope	0.019	0.010	0.000	0.039	0.048	0.433
EP intercept						
EP slope	-0.326	0.072	-0.466	-0.185	<0.001	-0.649
In the E/UE group						
IP intercept						
IP slope	-0.051	0.090	-0.227	0.125	0.567	-0.104
EP intercept	1.539	0.559	0.443	2.634	0.006	0.326
EP slope	-0.011	0.070	-0.149	0.127	0.875	-0.024
IP slope						
EP intercept	0.050	0.071	-0.088	0.188	0.479	0.108
EP slope	0.008	0.013	-0.017	0.033	0.542	0.173
EP intercept						
EP slope	-0.170	0.077	-0.320	-0.019	0.027	-0.397
In the HE group						
IP intercept						
IP slope	-0.128	0.054	-0.234	-0.021	0.018	-0.279
EP intercept	0.664	0.329	0.019	1.310	0.044	0.177
EP slope	-0.017	0.040	-0.095	0.062	0.679	-0.047
IP slope						
EP intercept	0.088	0.048	-0.006	0.181	0.065	0.176
EP slope	0.019	0.008	0.003	0.035	0.021	0.400
EP intercept						
EP slope	-0.110	0.048	-0.204	-0.015	0.023	-0.284

Supplementary Table S6. Differences in GMV development between groups.

Contrast	Cluster level		Peak level				Brain area (AAL3)	
	p_{FWE}	k_E	F	MNI (mm)				
				x	y	z	hemisphere	brain region
Age by group interaction: RE versus HE								
	< 0.001	1476	23.07	-16	-62	-60	left	Cerebelum_8
			18.11	-9	-64	-45	left	Cerebelum_8
			16.58	0	-57	-56	left	Cerebelum_9
Age by group interaction: E/UE versus HE								
	< 0.001	3434	35.25	-15	-63	-57	left	Cerebelum_8
			30.81	-8	-66	-42	left	Cerebelum_8
			25.76	-9	-64	-51	left	Cerebelum_8
	< 0.001	2069	24.83	-6	-54	-4	left	Cerebelum_4_5
			18.95	-14	-66	0	left	Lingual
			18.73	-9	-45	-10	left	Cerebelum_4_5
	< 0.001	1669	24.74	48	33	28	right	Frontal_Mid_2
			24.42	46	27	36	right	Frontal_Mid_2
			21.15	39	28	42	right	Frontal_Mid_2
	< 0.001	1087	22.83	34	-10	2	right	Putamen
			22.04	34	-2	4	right	Putamen
			16.51	36	8	-4	right	Putamen
	< 0.001	764	27.56	10	68	-2	right	Frontal_Sup_Medial
			18.28	12	68	9	right	Frontal_Sup_Medial
			17.85	10	62	22	right	Frontal_Sup_Medial
	0.006	501	26.73	38	57	-9	right	Frontal_Mid_2
			21.56	44	51	-6	right	Frontal_Mid_2
			15.85	36	58	2	right	Frontal_Mid_2
	0.008	480	25.54	15	3	-32	right	ParaHippocampal
			17.50	26	0	-36	right	ParaHippocampal
			16.67	21	-6	-28	right	ParaHippocampal
	0.009	468	17.28	51	-24	56	right	Postcentral
			16.30	54	-32	52	right	Parietal_Inf
			15.27	40	-38	52	right	Postcentral

Supplementary Table S7. The age-by-group interactions of GMV changes among different groups using the mixed linear models. RE, restrictive eaters; E/UE, emotional and uncontrolled eaters; HE, healthy eaters. The primary analysis was adjusted for sex, recruitment sites, total intracranial volume.

The age-by-group interaction in the RE and HE groups					
ROI	hemisphere	beta	se	t	p
In the primary analysis					
Cerebelum_8	left	-0.023	0.0047	-4.91	1E-06
Adjusted for pubertal status					
Cerebelum_8	left	-0.024	0.005	-4.75	3E-06
Adjusted for IQ					
Cerebelum_8	left	-0.024	0.0049	-4.86	1E-06
Adjusted for educational attainment					
Cerebelum_8	left	-0.024	0.0047	-5.02	7E-07
Adjusted for age- and sex-adjusted BMI-Zscore					
Cerebelum_8	left	-0.023	0.005	-4.57	6E-06
Adjusted for all above covariates					
Cerebelum_8	left	-0.023	0.0054	-4.27	2E-05

The age-by-group interaction in the E/UE and HE groups					
ROI	hemisphere	beta	se	t	p
In the primary analysis					
Cerebelum_8	left	-0.029	0.005	-5.8	1E-08
Frontal_Sup_Medial	right	-0.013	0.0026	-5	7E-07
Frontal_Mid_2	right	-0.014	0.0028	-4.87	1E-06
ParaHippocampal	right	0.0191	0.0038	5.035	6E-07
Cerebelum_4_5	left	-0.016	0.0031	-4.97	8E-07
Frontal_Mid_2	right	-0.013	0.0025	-5.21	3E-07
Putamen	right	-0.017	0.0037	-4.63	5E-06
Postcentral	right	-0.013	0.0028	-4.67	4E-06
Adjusted for pubertal status					
Cerebelum_8	left	-0.03	0.0054	-5.57	4E-08
Frontal_Sup_Medial	right	-0.014	0.0028	-5.17	3E-07
Frontal_Mid_2	right	-0.014	0.003	-4.8	2E-06
ParaHippocampal	right	0.0199	0.0042	4.787	2E-06
Cerebelum_4_5	left	-0.016	0.0034	-4.7	3E-06
Frontal_Mid_2	right	-0.015	0.0027	-5.62	3E-08
Putamen	right	-0.018	0.0041	-4.33	2E-05
Postcentral	right	-0.014	0.003	-4.6	5E-06
Adjusted for IQ					
Cerebelum_8	left	-0.033	0.0052	-6.41	3E-10
Frontal_Sup_Medial	right	-0.013	0.0027	-4.8	2E-06
Frontal_Mid_2	right	-0.014	0.0029	-4.71	3E-06
ParaHippocampal	right	0.0189	0.0039	4.914	1E-06
Cerebelum_4_5	left	-0.016	0.0032	-5.11	4E-07
Frontal_Mid_2	right	-0.012	0.0026	-4.45	1E-05
Putamen	right	-0.017	0.0039	-4.28	2E-05

Postcentral	right	-0.011	0.0029	-3.86	1E-04
Adjusted for educational attainment					
Cerebelum_8	left	-0.029	0.005	-5.75	1E-08
Frontal_Sup_Medial	right	-0.012	0.0026	-4.74	3E-06
Frontal_Mid_2	right	-0.013	0.0028	-4.73	3E-06
ParaHippocampal	right	0.0194	0.0039	5.011	7E-07
Cerebelum_4_5	left	-0.015	0.0032	-4.82	2E-06
Frontal_Mid_2	right	-0.013	0.0025	-5.04	6E-07
Putamen	right	-0.016	0.0038	-4.38	1E-05
Postcentral	right	-0.013	0.0028	-4.52	7E-06
Adjusted for age- and sex-adjusted BMI-Zscore					
Cerebelum_8	left	-0.027	0.0052	-5.28	2E-07
Frontal_Sup_Medial	right	-0.011	0.0027	-4.22	3E-05
Frontal_Mid_2	right	-0.013	0.0029	-4.39	1E-05
ParaHippocampal	right	0.021	0.004	5.271	2E-07
Cerebelum_4_5	left	-0.015	0.0033	-4.54	7E-06
Frontal_Mid_2	right	-0.012	0.0026	-4.46	1E-05
Putamen	right	-0.016	0.0039	-4.04	6E-05
Postcentral	right	-0.012	0.0029	-4.25	2E-05
Adjusted for all above covariates					
Cerebelum_8	left	-0.032	0.0057	-5.54	5E-08
Frontal_Sup_Medial	right	-0.015	0.003	-5.01	8E-07
Frontal_Mid_2	right	-0.016	0.0033	-4.84	2E-06
ParaHippocampal	right	0.0207	0.0042	4.916	1E-06
Cerebelum_4_5	left	-0.017	0.0035	-4.85	2E-06
Frontal_Mid_2	right	-0.015	0.0029	-5.03	7E-07
Putamen	right	-0.017	0.0043	-3.98	8E-05
Postcentral	right	-0.013	0.0032	-4.17	4E-05

No significant regions displaying age-by-group interactions between the RE and E/UE groups were identified in the primary VBM analysis.

Supplementary Table S8. The age-by-group interactions of CT changes among different groups using the mixed linear models. RE, restrictive eaters; E/UE, emotional and uncontrolled eaters; HE, healthy eaters. The analyses were adjusted for sex, recruitment sites, total intracranial volume.

The age-by-group interaction in the RE and E/UE groups							The age-by-group interaction in the RE and HE groups							The age-by-group interaction in the E/UE and HE groups						
ROI	hemisphere	beta	se	t	p	$p_{Bonferroni}$	ROI	hemisphere	beta	se	t	p	$p_{Bonferroni}$	ROI	hemisphere	beta	se	t	p	$p_{Bonferroni}$
bankssts	left	0.030	0.017	1.798	0.073	1	bankssts	left	1E-06	0.007	2E-04	1	1	bankssts	left	-0.029	0.013	-2.24	0.026	1
bankssts	right	0.027	0.012	2.229	0.026	1	bankssts	right	0.0049	0.005	0.941	0.347	1	bankssts	right	-0.017	0.010	-1.67	0.096	1
caudalanteriorcingulate	left	0.012	0.015	0.792	0.429	1	caudalanteriorcingulate	left	-0.007	0.006	-1.13	0.258	1	caudalanteriorcingulate	left	-0.026	0.012	-2.17	0.031	1
caudalanteriorcingulate	right	0.037	0.017	2.23	0.026	1	caudalanteriorcingulate	right	-0.007	0.007	-1.02	0.306	1	caudalanteriorcingulate	right	-0.052	0.014	-3.8	2E-04	0.03268
caudalmiddlefrontal	left	0.008	0.012	0.609	0.543	1	caudalmiddlefrontal	left	-0.01	0.005	-1.89	0.059	1	caudalmiddlefrontal	left	-0.027	0.011	-2.47	0.014	1
caudalmiddlefrontal	right	0.020	0.013	1.592	0.112	1	caudalmiddlefrontal	right	-0.013	0.006	-2.35	0.019	1	caudalmiddlefrontal	right	-0.046	0.012	-3.9	1E-04	0.02186
cuneus	left	0.024	0.010	2.535	0.012	1	cuneus	left	-0.005	0.004	-1.15	0.252	1	cuneus	left	-0.033	0.008	-4.11	5E-05	0.00923
cuneus	right	0.024	0.010	2.459	0.014	1	cuneus	right	0.0002	0.004	0.047	0.963	1	cuneus	right	-0.023	0.009	-2.59	0.01	1
entorhinal	left	-0.048	0.030	-1.61	0.109	1	entorhinal	left	-0.002	0.013	-0.15	0.882	1	entorhinal	left	0.0438	0.028	1.574	0.116	1
entorhinal	right	-0.098	0.035	-2.79	0.006	1	entorhinal	right	-0.011	0.015	-0.7	0.486	1	entorhinal	right	0.0762	0.033	2.338	0.02	1
fusiform	left	0.011	0.012	0.928	0.354	1	fusiform	left	0.0009	0.005	0.163	0.87	1	fusiform	left	-0.009	0.012	-0.82	0.412	1
fusiform	right	0.012	0.012	1.037	0.3	1	fusiform	right	-2E-04	0.005	-0.05	0.964	1	fusiform	right	-0.012	0.012	-1.07	0.286	1
inferiorparietal	left	0.009	0.012	0.784	0.434	1	inferiorparietal	left	-0.006	0.005	-1.36	0.175	1	inferiorparietal	left	-0.022	0.010	-2.11	0.035	1
inferiorparietal	right	0.018	0.010	1.773	0.077	1	inferiorparietal	right	-0.004	0.004	-0.93	0.353	1	inferiorparietal	right	-0.026	0.009	-2.8	0.005	1
inferiortemporal	left	0.012	0.012	1.058	0.291	1	inferiortemporal	left	0.0024	0.005	0.454	0.65	1	inferiortemporal	left	-0.008	0.010	-0.72	0.474	1
inferiortemporal	right	0.022	0.011	1.974	0.049	1	inferiortemporal	right	0.0061	0.005	1.201	0.23	1	inferiortemporal	right	-0.01	0.011	-0.9	0.368	1
isthmuscingulate	left	0.018	0.012	1.527	0.127	1	isthmuscingulate	left	-0.005	0.006	-0.95	0.342	1	isthmuscingulate	left	-0.028	0.012	-2.46	0.014	1
isthmuscingulate	right	0.020	0.010	2.127	0.034	1	isthmuscingulate	right	-0.002	0.005	-0.36	0.718	1	isthmuscingulate	right	-0.024	0.010	-2.29	0.023	1
lateraloccipital	left	0.027	0.009	2.86	0.004	0.89815	lateraloccipital	left	-1E-04	0.004	-0.03	0.978	1	lateraloccipital	left	-0.027	0.009	-3.03	0.003	0.52519

lateraloccipital	right	0.019	0.010	2.025	0.043	1	lateraloccipital	right	-0.003	0.004	-0.82	0.415	1	lateraloccipital	right	-0.026	0.009	-2.92	0.004	0.74065
lateralorbitofrontal	left	0.012	0.014	0.872	0.383	1	lateralorbitofrontal	left	0.0002	0.006	0.026	0.979	1	lateralorbitofrontal	left	-0.011	0.012	-0.91	0.361	1
lateralorbitofrontal	right	0.016	0.013	1.168	0.243	1	lateralorbitofrontal	right	0.0011	0.006	0.196	0.844	1	lateralorbitofrontal	right	-0.013	0.012	-1.11	0.267	1
lingual	left	0.040	0.012	3.253	0.001	0.2477	lingual	left	-0.008	0.006	-1.4	0.161	1	lingual	left	-0.055	0.011	-5.09	5E-07	9.6E-05
lingual	right	0.021	0.010	2.142	0.033	1	lingual	right	-8E-05	0.005	-0.02	0.987	1	lingual	right	-0.021	0.010	-2.03	0.042	1
medialorbitofrontal	left	0.015	0.012	1.289	0.198	1	medialorbitofrontal	left	0.001	0.005	0.207	0.836	1	medialorbitofrontal	left	-0.013	0.011	-1.25	0.211	1
medialorbitofrontal	right	0.013	0.013	1.056	0.292	1	medialorbitofrontal	right	0.0037	0.006	0.645	0.519	1	medialorbitofrontal	right	-0.006	0.012	-0.48	0.63	1
middletemporal	left	0.023	0.012	1.88	0.061	1	middletemporal	left	0.0011	0.005	0.208	0.835	1	middletemporal	left	-0.02	0.011	-1.93	0.054	1
middletemporal	right	0.024	0.010	2.411	0.016	1	middletemporal	right	-0.003	0.005	-0.57	0.567	1	middletemporal	right	-0.029	0.010	-3.01	0.003	0.55234
parahippocampal	left	0.011	0.015	0.738	0.461	1	parahippocampal	left	-0.003	0.007	-0.38	0.704	1	parahippocampal	left	-0.016	0.014	-1.18	0.239	1
parahippocampal	right	-0.003	0.017	-0.21	0.837	1	parahippocampal	right	-0.009	0.007	-1.26	0.21	1	parahippocampal	right	-0.015	0.016	-0.93	0.354	1
paracentral	left	0.002	0.010	0.151	0.88	1	paracentral	left	-0.013	0.005	-2.92	0.004	0.73028	paracentral	left	-0.028	0.010	-2.83	0.005	0.97225
paracentral	right	0.002	0.011	0.203	0.84	1	paracentral	right	-0.009	0.004	-1.9	0.058	1	paracentral	right	-0.019	0.009	-2.06	0.04	1
parsopercularis	left	0.006	0.010	0.575	0.565	1	parsopercularis	left	-9E-04	0.005	-0.18	0.857	1	parsopercularis	left	-0.007	0.010	-0.74	0.46	1
parsopercularis	right	0.014	0.010	1.44	0.15	1	parsopercularis	right	-0.008	0.005	-1.84	0.066	1	parsopercularis	right	-0.031	0.010	-3.26	0.001	0.23888
parsorbitalis	left	0.017	0.015	1.143	0.254	1	parsorbitalis	left	0.0029	0.006	0.458	0.647	1	parsorbitalis	left	-0.011	0.013	-0.87	0.387	1
parsorbitalis	right	-0.001	0.014	-0.07	0.947	1	parsorbitalis	right	-0.008	0.006	-1.27	0.203	1	parsorbitalis	right	-0.015	0.013	-1.15	0.253	1
parstriangularis	left	0.004	0.011	0.38	0.704	1	parstriangularis	left	-0.003	0.004	-0.78	0.435	1	parstriangularis	left	-0.011	0.009	-1.18	0.237	1
parstriangularis	right	0.012	0.011	1.071	0.284	1	parstriangularis	right	-0.008	0.005	-1.57	0.118	1	parstriangularis	right	-0.026	0.010	-2.7	0.007	1
pericalcarine	left	0.022	0.012	1.834	0.067	1	pericalcarine	left	-0.012	0.005	-2.27	0.024	1	pericalcarine	left	-0.045	0.010	-4.47	9E-06	0.00185
pericalcarine	right	0.015	0.011	1.402	0.161	1	pericalcarine	right	-0.007	0.005	-1.49	0.138	1	pericalcarine	right	-0.029	0.010	-2.81	0.005	1
postcentral	left	0.007	0.009	0.765	0.445	1	postcentral	left	-0.006	0.004	-1.74	0.082	1	postcentral	left	-0.019	0.008	-2.44	0.015	1

postcentral	right	0.012	0.009	1.295	0.196	1	postcentral	right	-0.008	0.004	-2.23	0.026	1	postcentral	right	-0.029	0.008	-3.42	7E-04	0.13608
posteriorcingulate	left	-0.006	0.011	-0.52	0.607	1	posteriorcingulate	left	-0.007	0.005	-1.56	0.118	1	posteriorcingulate	left	-0.009	0.008	-1.09	0.277	1
posteriorcingulate	right	0.003	0.008	0.41	0.682	1	posteriorcingulate	right	-0.003	0.004	-0.76	0.447	1	posteriorcingulate	right	-0.009	0.008	-1.16	0.248	1
precentral	left	0.008	0.012	0.648	0.517	1	precentral	left	-0.007	0.005	-1.32	0.186	1	precentral	left	-0.021	0.010	-2.01	0.045	1
precentral	right	0.010	0.011	0.838	0.402	1	precentral	right	-0.012	0.005	-2.57	0.01	1	precentral	right	-0.033	0.010	-3.25	0.001	0.24768
precuneus	left	0.016	0.009	1.65	0.1	1	precuneus	left	-0.006	0.004	-1.49	0.137	1	precuneus	left	-0.027	0.009	-3.04	0.002	0.50767
precuneus	right	0.006	0.009	0.661	0.509	1	precuneus	right	-0.004	0.004	-1	0.317	1	precuneus	right	-0.014	0.009	-1.63	0.104	1
rostralanteriorcingulate	left	0.013	0.012	1.077	0.282	1	rostralanteriorcingulate	left	0.0021	0.005	0.394	0.694	1	rostralanteriorcingulate	left	-0.009	0.011	-0.78	0.435	1
rostralanteriorcingulate	right	0.045	0.015	3.068	0.002	0.46129	rostralanteriorcingulate	right	0.0066	0.007	1.001	0.317	1	rostralanteriorcingulate	right	-0.031	0.013	-2.42	0.016	1
rostralmiddlefrontal	left	0.025	0.011	2.338	0.02	1	rostralmiddlefrontal	left	-0.007	0.005	-1.43	0.154	1	rostralmiddlefrontal	left	-0.038	0.010	-3.88	1E-04	0.02333
rostralmiddlefrontal	right	0.026	0.012	2.145	0.032	1	rostralmiddlefrontal	right	-0.012	0.005	-2.23	0.026	1	rostralmiddlefrontal	right	-0.049	0.011	-4.54	7E-06	0.00138
superiorfrontal	left	0.015	0.011	1.359	0.175	1	superiorfrontal	left	-0.01	0.005	-2.06	0.04	1	superiorfrontal	left	-0.035	0.010	-3.32	1E-03	0.19617
superiorfrontal	right	0.020	0.011	1.78	0.076	1	superiorfrontal	right	-0.009	0.005	-1.87	0.062	1	superiorfrontal	right	-0.038	0.011	-3.59	4E-04	0.07239
superiorparietal	left	0.006	0.011	0.528	0.598	1	superiorparietal	left	-0.005	0.005	-1.09	0.276	1	superiorparietal	left	-0.015	0.010	-1.55	0.121	1
superiorparietal	right	0.009	0.011	0.868	0.386	1	superiorparietal	right	-0.006	0.004	-1.29	0.199	1	superiorparietal	right	-0.02	0.010	-2.09	0.037	1
superiortemporal	left	0.023	0.011	2.078	0.038	1	superiortemporal	left	0.0006	0.005	0.114	0.91	1	superiortemporal	left	-0.022	0.010	-2.13	0.034	1
superiortemporal	right	0.034	0.012	2.879	0.004	0.84517	superiortemporal	right	-0.001	0.005	-0.24	0.807	1	superiortemporal	right	-0.036	0.011	-3.22	0.001	0.27515
supramarginal	left	0.009	0.011	0.809	0.419	1	supramarginal	left	-0.006	0.005	-1.36	0.175	1	supramarginal	left	-0.021	0.010	-2	0.046	1
supramarginal	right	0.017	0.010	1.781	0.076	1	supramarginal	right	-0.007	0.004	-1.84	0.066	1	supramarginal	right	-0.032	0.009	-3.61	3E-04	0.06645
frontalpole	left	0.062	0.021	2.882	0.004	0.83931	frontalpole	left	-0.016	0.009	-1.73	0.084	1	frontalpole	left	-0.094	0.019	-5.05	6E-07	0.00012
frontalpole	right	0.070	0.019	3.701	2E-04	0.0483	frontalpole	right	0.0009	0.009	0.101	0.92	1	frontalpole	right	-0.068	0.018	-3.76	2E-04	0.03759

temporalpole	left	-0.021	0.041	-0.51	0.613	1	temporalpole	left	-0.014	0.018	-0.79	0.432	1	temporalpole	left	-0.007	0.037	-0.18	0.857	1
temporalpole	right	-0.043	0.043	-1	0.317	1	temporalpole	right	-0.002	0.018	-0.12	0.908	1	temporalpole	right	0.039	0.040	0.987	0.324	1
transversetemporal	left	0.031	0.016	1.94	0.053	1	transversetemporal	left	-0.009	0.006	-1.37	0.171	1	transversetemporal	left	-0.048	0.015	-3.26	0.001	0.24086
transversetemporal	right	0.044	0.017	2.575	0.01	1	transversetemporal	right	0.0033	0.007	0.482	0.63	1	transversetemporal	right	-0.037	0.015	-2.44	0.015	1
insula	left	0.039	0.019	2.044	0.041	1	insula	left	-2E-04	0.008	-0.03	0.979	1	insula	left	-0.039	0.018	-2.12	0.034	1
insula	right	0.052	0.020	2.58	0.01	1	insula	right	-4E-04	0.009	-0.04	0.968	1	insula	right	-0.052	0.019	-2.71	0.007	1

Supplementary Table S9. The age-by-group interactions of CT changes among different groups using the mixed linear models, accounting for the effects of pubertal status, IQ, educational attainment, and age- and sex-adjusted BMI Z-score, and Euler's numbers. RE, restrictive eaters; E/UE, emotional and uncontrolled eaters; HE, healthy eaters. [a]The column "remain sig.' indicates whether the primary analyses remain Bonferroni significant (p < 0.05/(68*3) = 2.45×10-4) after adjusting for the corresponding covariates.

The age-by-group interaction in the RE and E/UE groups						
ROI	hemisphere	beta	se	t	p	remain sig.[a]
In the primary analysis (N of significant ROIs = 1)						
frontalpole	right	0.0697	0.0188	3.701	0.00024	
Adjusted for pubertal status						
frontalpole	right	0.0767	0.0202	3.794	0.00017	sig.
Adjusted for IQ						
frontalpole	right	0.074	0.0198	3.736	0.00021	sig.
Adjusted for educational attainment						
frontalpole	right	0.0673	0.0188	3.575	0.00038	
Adjusted for age- and sex-adjusted BMI-Zscore						
frontalpole	right	0.0644	0.02	3.219	0.00137	
Adjusted for Euler's number						
frontalpole	right	0.0696	0.0188	3.696	0.00024	sig.
Adjusted for all above covariates						
frontalpole	right	0.0764	0.0223	3.422	0.00068	

The age-by-group interaction in the E/UE and HE groups						
ROI	hemisphere	beta	se	t	p	remain sig.[a]
In the primary analysis (N of significant ROIs = 9)						
lingual	left	-0.055	0.0108	-5.09	4.7E-07	
frontalpole	left	-0.094	0.0187	-5.05	5.9E-07	
rostralmiddlefrontal	right	-0.049	0.0107	-4.54	6.8E-06	
pericalcarine	left	-0.045	0.0101	-4.47	9.1E-06	
cuneus	left	-0.033	0.0081	-4.11	4.5E-05	
caudalmiddlefrontal	right	-0.046	0.0119	-3.9	0.00011	
rostralmiddlefrontal	left	-0.038	0.0098	-3.88	0.00011	
caudalanteriorcingulate	right	-0.052	0.0136	-3.8	0.00016	
frontalpole	right	-0.068	0.0181	-3.76	0.00018	
Ajusted for pubertal status						
lingual	left	-0.059	0.0114	-5.21	2.7E-07	sig.
frontalpole	left	-0.099	0.0201	-4.94	1E-06	sig.
rostralmiddlefrontal	right	-0.057	0.0116	-4.92	1.1E-06	sig.
pericalcarine	left	-0.051	0.0106	-4.84	1.7E-06	sig.
cuneus	left	-0.038	0.0086	-4.47	9.6E-06	sig.
caudalmiddlefrontal	right	-0.058	0.0128	-4.49	8.7E-06	sig.
rostralmiddlefrontal	left	-0.048	0.0106	-4.52	7.5E-06	sig.
caudalanteriorcingulate	right	-0.053	0.0142	-3.72	0.00022	sig.
frontalpole	right	-0.072	0.0195	-3.71	0.00023	sig.
Adjusted for IQ						
lingual	left	-0.061	0.0112	-5.41	9.1E-08	sig.
frontalpole	left	-0.104	0.0191	-5.44	8E-08	sig.
rostralmiddlefrontal	right	-0.048	0.0112	-4.33	1.8E-05	sig.

pericalcarine	left	-0.045	0.0105	-4.24	2.6E-05	sig.
cuneus	left	-0.037	0.0083	-4.39	1.3E-05	sig.
caudalmiddlefrontal	right	-0.046	0.0121	-3.83	0.00014	sig.
rostralmiddlefrontal	left	-0.043	0.0103	-4.16	3.7E-05	sig.
caudalanteriorcingulate	right	-0.054	0.0146	-3.69	0.00025	sig.
frontalpole	right	-0.075	0.0184	-4.08	5.2E-05	sig.
Adjusted for educational attainment						
lingual	left	-0.056	0.0109	-5.13	3.8E-07	sig.
frontalpole	left	-0.095	0.0187	-5.1	4.4E-07	sig.
rostralmiddlefrontal	right	-0.05	0.0107	-4.65	4.1E-06	sig.
pericalcarine	left	-0.045	0.0102	-4.46	9.5E-06	sig.
cuneus	left	-0.033	0.0081	-4.11	4.5E-05	sig.
caudalmiddlefrontal	right	-0.047	0.0119	-3.99	7.4E-05	sig.
rostralmiddlefrontal	left	-0.039	0.0098	-3.99	7.4E-05	sig.
caudalanteriorcingulate	right	-0.051	0.0137	-3.71	0.00022	sig.
frontalpole	right	-0.068	0.0181	-3.75	0.00019	sig.
Adjusted for age- and sex-adjusted BMI-Zscore						
lingual	left	-0.053	0.0114	-4.67	3.7E-06	sig.
frontalpole	left	-0.091	0.0193	-4.73	2.8E-06	sig.
rostralmiddlefrontal	right	-0.047	0.0112	-4.24	2.6E-05	sig.
pericalcarine	left	-0.04	0.0105	-3.82	0.00015	sig.
cuneus	left	-0.031	0.0085	-3.59	0.00036	
caudalmiddlefrontal	right	-0.041	0.0123	-3.33	0.00093	
rostralmiddlefrontal	left	-0.037	0.0102	-3.65	0.00029	
caudalanteriorcingulate	right	-0.047	0.0142	-3.28	0.00109	
frontalpole	right	-0.068	0.0182	-3.74	0.00021	sig.
Adjusted for Euler's number						
lingual	left	-0.055	0.0108	-5.09	4.8E-07	sig.
frontalpole	left	-0.094	0.0187	-5.04	6E-07	sig.
rostralmiddlefrontal	right	-0.049	0.0107	-4.53	7E-06	sig.
pericalcarine	left	-0.045	0.0101	-4.47	9.2E-06	sig.

cuneus	left	-0.033	0.0081	-4.1	4.6E-05	sig.
caudalmiddlefrontal	right	-0.046	0.0119	-3.89	0.00011	sig.
rostralmiddlefrontal	left	-0.038	0.0098	-3.87	0.00012	sig.
caudalanteriorcingulate	right	-0.052	0.0136	-3.79	0.00016	sig.
frontalpole	right	-0.068	0.0181	-3.76	0.00019	sig.
Adjusted for all above covariates						
lingual	left	-0.062	0.0121	-5.16	3.6E-07	sig.
frontalpole	left	-0.1	0.0209	-4.79	2.2E-06	sig.
rostralmiddlefrontal	right	-0.055	0.0125	-4.4	1.3E-05	sig.
pericalcarine	left	-0.05	0.0113	-4.43	1.2E-05	sig.
cuneus	left	-0.041	0.009	-4.51	8.1E-06	sig.
caudalmiddlefrontal	right	-0.053	0.0136	-3.9	0.00011	sig.
rostralmiddlefrontal	left	-0.049	0.0113	-4.31	2E-05	sig.
caudalanteriorcingulate	right	-0.052	0.0154	-3.36	0.00084	
frontalpole	right	-0.072	0.02	-3.6	0.00035	

No significant age by group interaction were found between the RE and HE groups in the primary analysis.

Supplementary Table S10. The age-by-group interactions of SD changes among different groups using the mixed linear models. RE, restrictive eaters; E/UE, emotional and uncontrolled eaters; HE, healthy eaters. The analyses were adjusted for sex, recruitment sites, total intracranial volume.

The age-by-group interaction in the RE and E/UE groups							The age-by-group interaction in the RE and HE groups							The age-by-group interaction in the E/UE and HE groups						
ROI	hemisphere	beta	se	t	p	$p_{Bonferroni}$	ROI	hemisphere	beta	se	t	p	$p_{Bonferroni}$	ROI	hemisphere	beta	se	t	p	$p_{Bonferroni}$
bankssts	left	0.0027	0.0054	0.502	0.616	1	bankssts	left	0.0036	0.0025	1.47	0.142	1	bankssts	left	0.0045	0.0049	0.92	0.355	1
bankssts	right	-0.007	0.0056	-1.29	0.198	1	bankssts	right	0.0038	0.0026	1.44	0.15	1	bankssts	right	0.015	0.005	2.97	0.003	0.62597
caudalanteriorcingulate	left	0.0012	0.0077	0.149	0.882	1	caudalanteriorcingulate	left	-0.001	0.0034	-0.40	0.688	1	caudalanteriorcingulate	left	-0.004	0.0065	-0.61	0.539	1
caudalanteriorcingulate	right	-0.002	0.008	-0.29	0.775	1	caudalanteriorcingulate	right	-0.007	0.0033	-2.12	0.035	1	caudalanteriorcingulate	right	-0.012	0.0064	-1.82	0.069	1
caudalmiddlefrontal	left	-0.019	0.0059	-3.22	0.001	0.27619	caudalmiddlefrontal	left	0.0003	0.0028	0.11	0.916	1	caudalmiddlefrontal	left	0.0195	0.0058	3.37	8E-04	0.16199
caudalmiddlefrontal	right	-0.011	0.0073	-1.54	0.125	1	caudalmiddlefrontal	right	0.0072	0.0032	2.27	0.024	1	caudalmiddlefrontal	right	0.0258	0.0062	4.15	4E-05	0.00756
cuneus	left	-0.008	0.0065	-1.19	0.235	1	cuneus	left	-0.002	0.0029	-0.81	0.419	1	cuneus	left	0.0032	0.0055	0.58	0.564	1
cuneus	right	-0.009	0.0051	-1.79	0.073	1	cuneus	right	-0.006	0.0026	-2.29	0.022	1	cuneus	right	-0.003	0.0055	-0.50	0.617	1
entorhinal	left	-0.024	0.0143	-1.7	0.09	1	entorhinal	left	-0.007	0.0064	-1.06	0.29	1	entorhinal	left	0.0106	0.014	0.75	0.452	1
entorhinal	right	0.0158	0.0147	1.073	0.284	1	entorhinal	right	-0.006	0.0065	-0.91	0.365	1	entorhinal	right	-0.027	0.0138	-1.99	0.047	1
fusiform	left	-0.013	0.0049	-2.58	0.01	1	fusiform	left	-0.002	0.0023	-0.77	0.443	1	fusiform	left	0.0091	0.0048	1.90	0.058	1
fusiform	right	-0.002	0.0054	-0.3	0.762	1	fusiform	right	-0.001	0.0024	-0.61	0.539	1	fusiform	right	-0.001	0.0051	-0.27	0.79	1
inferiorparietal	left	-0.009	0.0061	-1.51	0.132	1	inferiorparietal	left	0.0019	0.0027	0.70	0.482	1	inferiorparietal	left	0.0132	0.0056	2.34	0.019	1
inferiorparietal	right	-0.005	0.006	-0.8	0.421	1	inferiorparietal	right	0.0048	0.0026	1.86	0.064	1	inferiorparietal	right	0.0146	0.0057	2.56	0.011	1
inferiortemporal	left	-0.005	0.0055	-0.92	0.36	1	inferiortemporal	left	-4E-04	0.0023	-0.17	0.867	1	inferiortemporal	left	0.0044	0.0045	0.98	0.329	1
inferiortemporal	right	-0.007	0.0048	-1.52	0.129	1	inferiortemporal	right	-6E-04	0.0024	-0.24	0.807	1	inferiortemporal	right	0.0061	0.005	1.23	0.221	1
isthmuscingulate	left	0.0019	0.0051	0.372	0.71	1	isthmuscingulate	left	0.0026	0.0023	1.17	0.244	1	isthmuscingulate	left	0.0034	0.0044	0.76	0.447	1
isthmuscingulate	right	-0.007	0.0058	-1.13	0.259	1	isthmuscingulate	right	-0.001	0.0025	-0.52	0.605	1	isthmuscingulate	right	0.0041	0.0051	0.81	0.42	1
lateraloccipital	left	-0.008	0.0041	-2.02	0.044	1	lateraloccipital	left	0.0029	0.0017	1.66	0.098	1	lateraloccipital	left	0.0141	0.0039	3.58	4E-04	0.07422

lateraloccipital	right	-0.007	0.0043	-1.68	0.094	1	lateraloccipital	right	0.0033	0.0021	1.52	0.129	1	lateraloccipital	right	0.014	0.0044	3.15	0.002	0.34869
lateralorbitofrontal	left	-0.014	0.0064	-2.25	0.025	1	lateralorbitofrontal	left	0.002	0.003	0.68	0.497	1	lateralorbitofrontal	left	0.0182	0.0057	3.21	0.001	0.28527
lateralorbitofrontal	right	-0.007	0.0051	-1.44	0.15	1	lateralorbitofrontal	right	-3E-04	0.0024	-0.13	0.897	1	lateralorbitofrontal	right	0.0069	0.005	1.38	0.169	1
lingual	left	-0.006	0.0042	-1.53	0.128	1	lingual	left	0.0018	0.0019	0.96	0.34	1	lingual	left	0.0099	0.0043	2.31	0.021	1
lingual	right	0.0007	0.0045	0.164	0.87	1	lingual	right	0.0003	0.0022	0.15	0.879	1	lingual	right	-5E-05	0.0042	-0.01	0.991	1
medialorbitofrontal	left	-0.014	0.0081	-1.76	0.079	1	medialorbitofrontal	left	0.0046	0.0039	1.17	0.243	1	medialorbitofrontal	left	0.0235	0.0081	2.89	0.004	0.82355
medialorbitofrontal	right	-0.01	0.0079	-1.24	0.216	1	medialorbitofrontal	right	-0.005	0.0034	-1.46	0.145	1	medialorbitofrontal	right	8E-05	0.0071	0.01	0.991	1
middletemporal	left	-0.003	0.005	-0.65	0.518	1	middletemporal	left	0.0039	0.0022	1.81	0.071	1	middletemporal	left	0.0111	0.004	2.76	0.006	1
middletemporal	right	-0.009	0.0053	-1.74	0.082	1	middletemporal	right	0.0045	0.0027	1.65	0.1	1	middletemporal	right	0.0182	0.0055	3.27	0.001	0.22747
parahippocampal	left	-0.008	0.0069	-1.19	0.234	1	parahippocampal	left	-6E-04	0.003	-0.21	0.83	1	parahippocampal	left	0.0067	0.0066	1.03	0.305	1
parahippocampal	right	0.002	0.0061	0.319	0.75	1	parahippocampal	right	-0.001	0.0028	-0.48	0.632	1	parahippocampal	right	-0.005	0.006	-0.77	0.44	1
paracentral	left	-0.001	0.0065	-0.18	0.858	1	paracentral	left	0.001	0.0027	0.37	0.713	1	paracentral	left	0.0031	0.0059	0.52	0.606	1
paracentral	right	-0.015	0.0062	-2.36	0.019	1	paracentral	right	-0.006	0.0025	-2.41	0.016	1	paracentral	right	0.0028	0.0048	0.58	0.56	1
parsopercularis	left	-0.006	0.0055	-1.14	0.253	1	parsopercularis	left	0.0003	0.0025	0.13	0.897	1	parsopercularis	left	0.007	0.0046	1.52	0.129	1
parsopercularis	right	-0.012	0.0064	-1.9	0.059	1	parsopercularis	right	0.0043	0.0028	1.53	0.126	1	parsopercularis	right	0.0208	0.0052	3.97	8E-05	0.01605
parsorbitalis	left	-0.014	0.0077	-1.77	0.078	1	parsorbitalis	left	0.0058	0.0034	1.70	0.09	1	parsorbitalis	left	0.0251	0.0069	3.63	3E-04	0.06338
parsorbitalis	right	-0.014	0.0079	-1.82	0.069	1	parsorbitalis	right	0.0083	0.004	2.09	0.037	1	parsorbitalis	right	0.0309	0.0076	4.07	5E-05	0.01079
parstriangularis	left	-0.007	0.0061	-1.07	0.285	1	parstriangularis	left	0.0015	0.0026	0.56	0.575	1	parstriangularis	left	0.0095	0.0051	1.85	0.065	1
parstriangularis	right	-0.014	0.0072	-1.94	0.053	1	parstriangularis	right	0.0056	0.0033	1.69	0.092	1	parstriangularis	right	0.0252	0.0064	3.94	9E-05	0.01872
pericalcarine	left	0.0012	0.0056	0.205	0.838	1	pericalcarine	left	0.0031	0.0026	1.18	0.237	1	pericalcarine	left	0.005	0.0056	0.90	0.369	1
pericalcarine	right	-0.008	0.0057	-1.46	0.146	1	pericalcarine	right	-0.005	0.0028	-1.79	0.073	1	pericalcarine	right	-0.002	0.0055	-0.29	0.769	1

postcentral	left	-0.012	0.0069	-1.8	0.072	1	postcentral	left	0.0017	0.003	0.57	0.567	1	postcentral	left	0.0159	0.0059	2.68	0.007	1
postcentral	right	-0.01	0.0063	-1.58	0.114	1	postcentral	right	0.0043	0.0027	1.61	0.108	1	postcentral	right	0.0186	0.0056	3.32	1E-03	0.197 33
posteriorcing ulate	left	0.0032	0.0056	0.567	0.571	1	posteriorcin gulate	left	0.0001	0.0023	0.06	0.949	1	posteriorcing ulate	left	-0.003	0.0042	-0.70	0.482	1
posteriorcing ulate	right	-0.006	0.0063	-0.99	0.323	1	posteriorcin gulate	right	-0.005	0.0025	-1.82	0.069	1	posteriorcing ulate	right	-0.003	0.0054	-0.57	0.571	1
precentral	left	-0.011	0.0055	-2.07	0.038	1	precentral	left	0.0012	0.0025	0.49	0.621	1	precentral	left	0.014	0.0049	2.85	0.005	0.928 74
precentral	right	-0.011	0.0057	-2.01	0.045	1	precentral	right	0.0033	0.0025	1.29	0.197	1	precentral	right	0.0182	0.0051	3.54	4E-04	0.086
precuneus	left	-0.002	0.0053	-0.39	0.698	1	precuneus	left	0.0021	0.0023	0.93	0.354	1	precuneus	left	0.0061	0.0047	1.31	0.192	1
precuneus	right	-0.012	0.005	-2.35	0.019	1	precuneus	right	-0.005	0.0022	-2.25	0.025	1	precuneus	right	0.002	0.0043	0.46	0.648	1
rostralanterior cingulate	left	-0.01	0.0071	-1.45	0.148	1	rostralanteri orcingulate	left	0.0018	0.0035	0.53	0.597	1	rostralanterio rcingulate	left	0.0139	0.0074	1.87	0.062	1
rostralanterior cingulate	right	-0.004	0.0079	-0.47	0.64	1	rostralanteri orcingulate	right	-0.004	0.0035	-1.05	0.293	1	rostralanterio rcingulate	right	-0.004	0.0073	-0.48	0.631	1
rostralmiddlef rontal	left	-0.022	0.0072	-3.07	0.002	0.457 81	rostralmidd lefrontal	left	0.0061	0.0033	1.85	0.065	1	rostralmiddle frontal	left	0.0346	0.0068	5.11	4E-07	8.6E-05
rostralmiddlef rontal	right	-0.023	0.0074	-3.06	0.002	0.472 33	rostralmidd lefrontal	right	0.0086	0.0035	2.47	0.014	1	rostralmiddle frontal	right	0.04	0.0073	5.48	6E-08	1.2E-05
superiorfronta l	left	-0.015	0.0047	-3.09	0.002	0.434 06	superiorfro ntal	left	0.0049	0.0022	2.24	0.025	1	superiorfront al	left	0.0245	0.0047	5.19	3E-07	5.8E-05
superiorfronta l	right	-0.017	0.0053	-3.21	0.001	0.289 61	superiorfro ntal	right	0.002	0.0024	0.86	0.389	1	superiorfront al	right	0.021	0.0047	4.42	1E-05	0.002 4
superiorpariet al	left	-0.009	0.0066	-1.36	0.175	1	superiorpari etal	left	0.0017	0.0029	0.57	0.571	1	superiorparie tal	left	0.0123	0.0059	2.08	0.038	1
superiorpariet al	right	-0.01	0.0058	-1.74	0.083	1	superiorpari etal	right	0.0004	0.0024	0.15	0.883	1	superiorparie tal	right	0.0109	0.005	2.17	0.03	1
superiortemp oral	left	-0.004	0.0041	-1.09	0.278	1	superiortem poral	left	0.0026	0.0019	1.35	0.179	1	superiortemp oral	left	0.0097	0.0036	2.70	0.007	1
superiortemp oral	right	-0.008	0.0056	-1.4	0.163	1	superiortem poral	right	0.0038	0.0025	1.49	0.136	1	superiortemp oral	right	0.0156	0.0045	3.48	5E-04	0.110 74
supramargina l	left	-0.015	0.0074	-2	0.046	1	supramargi nal	left	0.0011	0.0032	0.34	0.735	1	supramargin al	left	0.0169	0.0065	2.60	0.009	1
supramargina l	right	-0.01	0.0064	-1.5	0.134	1	supramargi nal	right	0.0048	0.0027	1.76	0.078	1	supramargin al	right	0.0195	0.0061	3.18	0.002	0.310 96
frontalpole	left	-0.036	0.0096	-3.73	2E-04	0.043 22	frontalpole	left	0.0079	0.0044	1.78	0.075	1	frontalpole	left	0.0515	0.0094	5.47	6E-08	1.3E-05

frontalpole	right	-0.032	0.0108	-2.96	0.003	0.656 68	frontalpole	right	-0.009	0.0046	-1.86	0.063	1	frontalpole	right	0.015	0.0083	1.81	0.07	1
temporalpole	left	-0.019	0.0107	-1.77	0.078	1	temporalpol e	left	-0.002	0.0048	-0.45	0.655	1	temporalpole	left	0.0147	0.0106	1.38	0.167	1
temporalpole	right	0.0244	0.0122	2.009	0.045	1	temporalpol e	right	-0.003	0.0053	-0.61	0.54	1	temporalpole	right	-0.031	0.0115	-2.67	0.008	1
transversetem poral	left	-8E-04	0.0062	-0.13	0.897	1	transversete mporal	left	0.0003	0.0023	0.11	0.913	1	transversete mporal	left	0.0014	0.0044	0.31	0.754	1
transversetem poral	right	-0.01	0.0067	-1.52	0.129	1	transversete mporal	right	0.001	0.0028	0.36	0.719	1	transversete mporal	right	0.0125	0.0041	3.08	0.002	0.438 37
insula	left	-8E-04	0.0032	-0.25	0.803	1	insula	left	-0.002	0.0013	-1.40	0.161	1	insula	left	-0.003	0.0025	-1.09	0.278	1
insula	right	-0.005	0.004	-1.14	0.256	1	insula	right	-0.002	0.0017	-0.96	0.337	1	insula	right	0.0016	0.0026	0.64	0.524	1

Supplementary Table S11. The age-by-group interactions of SD changes among different groups using the mixed linear models, accounting for the effects of pubertal status, IQ, educational attainment, and age- and sex-adjusted BMI Z-score, and Euler's numbers. RE, restrictive eaters; E/UE, emotional and uncontrolled eaters; HE, healthy eaters. [a]The column "remain sig.' indicates whether the primary analyses remain Bonferroni significant ($p < 0.05/(68*3) = 2.45\times10^{-4}$) after adjusting for the corresponding covariates.

The age-by-group interaction in the RE and E/UE groups						
ROI	hemisphere	beta	se	t	p	remain sig.[a]
In the primary analysis (N of significant ROIs = 1)						
frontalpole	left	-0.036	0.0096	-3.73	0.00021	
Adjusted for pubertal status						
frontalpole	left	-0.029	0.0101	-2.88	0.00415	
Adjusted for IQ						
frontalpole	left	-0.036	0.0099	-3.63	0.00031	
Adjusted for educational attainment						
frontalpole	left	-0.035	0.0096	-3.68	0.00026	
Adjusted for age- and sex-adjusted BMI-Zscore						
frontalpole	left	-0.036	0.0102	-3.5	0.00051	
Adjusted for Euler's number						
frontalpole	left	-0.036	0.0096	-3.73	0.00021	sig.
Adjusted for all above covariates						
frontalpole	left	-0.031	0.0109	-2.88	0.00413	

The age-by-group interaction in the E/UE and HE groups						
ROI	hemisphere	beta	se	t	p	remain sig.[a]
In the primary analysis (N of significant ROIs = 9)						
rostralmiddlefrontal	right	0.04	0.0073	5.48	6.1E-08	
frontalpole	left	0.0515	0.0094	5.47	6.3E-08	
superiorfrontal	left	0.0245	0.0047	5.19	2.8E-07	
rostralmiddlefrontal	left	0.0346	0.0068	5.11	4.2E-07	
superiorfrontal	right	0.021	0.0047	4.42	1.2E-05	
caudalmiddlefrontal	right	0.0258	0.0062	4.15	3.7E-05	
parsorbitalis	right	0.0309	0.0076	4.07	5.3E-05	
parsopercularis	right	0.0208	0.0052	3.97	7.9E-05	
parstriangularis	right	0.0252	0.0064	3.94	9.2E-05	
Adjusted for pubertal status						
rostralmiddlefrontal	right	0.0393	0.0079	4.98	8.4E-07	sig.
frontalpole	left	0.0478	0.0098	4.87	1.5E-06	sig.
superiorfrontal	left	0.0251	0.0051	4.92	1.1E-06	sig.
rostralmiddlefrontal	left	0.0362	0.0072	5.00	7.5E-07	sig.
superiorfrontal	right	0.0216	0.0051	4.25	2.4E-05	sig.
caudalmiddlefrontal	right	0.0226	0.0067	3.36	0.00084	
parsorbitalis	right	0.0305	0.0081	3.75	0.00019	sig.
parsopercularis	right	0.0197	0.0055	3.60	0.00035	
parstriangularis	right	0.0267	0.0068	3.94	9E-05	sig.
Adjusted for IQ						
rostralmiddlefrontal	right	0.0397	0.0078	5.106	4.5E-07	sig.
frontalpole	left	0.0548	0.0095	5.734	1.6E-08	sig.
superiorfrontal	left	0.0242	0.005	4.794	2.1E-06	sig.
rostralmiddlefrontal	left	0.0344	0.0071	4.815	1.9E-06	sig.
superiorfrontal	right	0.0242	0.005	4.794	2.1E-06	sig.
caudalmiddlefrontal	right	0.0274	0.0066	4.152	3.8E-05	sig.

							parsorbitalis	right	0.0291	0.008	3.625	0.00031	
							parsopercularis	right	0.0212	0.0055	3.851	0.00013	sig.
							parstriangularis	right	0.0252	0.0068	3.693	0.00024	sig.
							Adjusted for educational attainment						
							rostralmiddlefrontal	right	0.0399	0.0073	5.438	7.7E-08	sig.
							frontalpole	left	0.0526	0.0093	5.663	2.3E-08	sig.
							superiorfrontal	left	0.0249	0.0047	5.258	2E-07	sig.
							rostralmiddlefrontal	left	0.0346	0.0068	5.097	4.6E-07	sig.
							superiorfrontal	right	0.0209	0.0047	4.409	1.2E-05	sig.
							caudalmiddlefrontal	right	0.0261	0.0062	4.183	3.3E-05	sig.
							parsorbitalis	right	0.0309	0.0076	4.06	5.5E-05	sig.
							parsopercularis	right	0.021	0.0053	3.993	7.3E-05	sig.
							parstriangularis	right	0.0254	0.0064	3.947	8.8E-05	sig.
							Adjusted for age- and sex-adjusted BMI-Zscore						
							rostralmiddlefrontal	right	0.0439	0.0077	5.695	1.9E-08	sig.
							frontalpole	left	0.0537	0.0099	5.436	8E-08	sig.
							superiorfrontal	left	0.0254	0.005	5.122	4.1E-07	sig.
							rostralmiddlefrontal	left	0.0384	0.0071	5.386	1E-07	sig.
							superiorfrontal	right	0.0227	0.005	4.54	6.8E-06	sig.
							caudalmiddlefrontal	right	0.0281	0.0065	4.315	1.9E-05	sig.
							parsorbitalis	right	0.0336	0.008	4.182	3.3E-05	sig.
							parsopercularis	right	0.0235	0.0055	4.279	2.2E-05	sig.
							parstriangularis	right	0.0281	0.0068	4.152	3.8E-05	sig.
							Adjusted for Euler's number						
							rostralmiddlefrontal	right	0.04	0.0073	5.48	6.1E-08	sig.
							frontalpole	left	0.0515	0.0094	5.472	6.4E-08	sig.
							superiorfrontal	left	0.0244	0.0047	5.187	2.9E-07	sig.
							rostralmiddlefrontal	left	0.0346	0.0068	5.113	4.2E-07	sig.
							superiorfrontal	right	0.0209	0.0047	4.411	1.2E-05	sig.
							caudalmiddlefrontal	right	0.0258	0.0062	4.152	3.7E-05	sig.
							parsorbitalis	right	0.0308	0.0076	4.068	5.3E-05	sig.
							parsopercularis	right	0.0208	0.0052	3.973	7.9E-05	sig.
							parstriangularis	right	0.0251	0.0064	3.933	9.3E-05	sig.
							Adjusted for all above covariates						
							rostralmiddlefrontal	right	0.042	0.0087	4.84	1.7E-06	sig.
							frontalpole	left	0.0513	0.0104	4.934	1.1E-06	sig.

							superiorfrontal	left	0.0278	0.0056	4.976	8.9E-07	sig.
							rostralmiddlefrontal	left	0.0391	0.0079	4.938	1.1E-06	sig.
							superiorfrontal	right	0.0257	0.0056	4.615	5E-06	sig.
							caudalmiddlefrontal	right	0.0245	0.0073	3.342	0.00089	
							parsorbitalis	right	0.0315	0.0089	3.559	0.00041	
							parsopercularis	right	0.0218	0.0059	3.667	0.00027	
							parstriangularis	right	0.0288	0.0074	3.883	0.00012	sig.

No significant age by group interaction were found between the RE and HE groups in the primary analysis.

References

1. Pritchard, J. K., Stephens, M. & Donnelly, P. Inference of population structure using multilocus genotype data. *Genetics* **155**, 945–959 (2000).
2. Purcell, S. *et al.* PLINK: a tool set for whole-genome association and population-based linkage analyses. *Am. J. Hum. Genet.* **81**, 559–575 (2007).
3. Price, A. L. *et al.* Principal components analysis corrects for stratification in genome-wide association studies. *Nat. Genet.* **38**, 904–909 (2006).
4. ENIGMA Genetics Support Team. ENIGMA 1KGP_p3v5 Cookbook [Online]. The Enhancing Neuroimaging Genetics through Meta-Analysis (ENIGMA) Consortium. (2017).
5. Hibar, D. P. *et al.* Common genetic variants influence human subcortical brain structures. *Nature* **520**, 224 (2015).
6. 1000 Genomes Project Consortium *et al.* A global reference for human genetic variation. *Nature* **526**, 68–74 (2015).
7. Manichaikul, A. *et al.* Robust relationship inference in genome-wide association studies. *Bioinforma. Oxf. Engl.* **26**, 2867–2873 (2010).
8. Conomos, M. P., Miller, M. B. & Thornton, T. A. Robust inference of population structure for ancestry prediction and correction of stratification in the presence of relatedness. *Genet. Epidemiol.* **39**, 276–293 (2015).
9. Das, S. *et al.* Next-generation genotype imputation service and methods. *Nat. Genet.* **48**, 1284–1287 (2016).
10. McNeish, D. & Wolf, M. G. Thinking twice about sum scores. *Behav. Res. Methods* **52**, 2287–2305 (2020).
