# Supplementary material for: Relationships of eating behaviors with psychopathology, brain maturation and genetic risk for obesity in an adolescent cohort study
Source: Nat Ment Health. 2025 Jan 10;3(1):58–70. doi: 10.1038/s44220-024-00354-7 (PMC11726452; doi:10.1038/s44220-024-00354-7)
Supplement: Supplementary file 2 — Reporting Summary [file 44220_2024_354_MOESM2_ESM.pdf]

nature portfolio

Corresponding author(s): Sylvane Desrivières

Last updated by author(s): Oct 4, 2024

Reporting Summary

Nature Portfolio wishes to improve the reproducibility of the work that we publish. This form provides structure for consistency and transparency in reporting. For further information on Nature Portfolio policies, see our Editorial Policies and the Editorial Policy Checklist.

Statistics

For all statistical analyses, confirm that the following items are present in the figure legend, table legend, main text, or Methods section.

n/a	Confirmed	
☐	☒	The exact sample size (*n*) for each experimental group/condition, given as a discrete number and unit of measurement
☐	☒	A statement on whether measurements were taken from distinct samples or whether the same sample was measured repeatedly
☐	☒	The statistical test(s) used AND whether they are one- or two-sided<br>*Only common tests should be described solely by name; describe more complex techniques in the Methods section.*
☐	☒	A description of all covariates tested
☐	☒	A description of any assumptions or corrections, such as tests of normality and adjustment for multiple comparisons
☐	☒	A full description of the statistical parameters including central tendency (e.g. means) or other basic estimates (e.g. regression coefficient) AND variation (e.g. standard deviation) or associated estimates of uncertainty (e.g. confidence intervals)
☐	☒	For null hypothesis testing, the test statistic (e.g. *F*, *t*, *r*) with confidence intervals, effect sizes, degrees of freedom and *P* value noted<br>*Give P values as exact values whenever suitable.*
☒	☐	For Bayesian analysis, information on the choice of priors and Markov chain Monte Carlo settings
☐	☒	For hierarchical and complex designs, identification of the appropriate level for tests and full reporting of outcomes
☐	☒	Estimates of effect sizes (e.g. Cohen's *d*, Pearson's *r*), indicating how they were calculated

Our web collection on statistics for biologists contains articles on many of the points above.

Software and code

Policy information about availability of computer code

Data collection	Delosis Psytools (version 1.0) was used for data collection: https://www.delosis.com/
Data analysis	No custom algorithms or software was developed for this study. Software and code for the neuroimaging analysis and data visualisation can be found at:<br>CAT 12.8 r1907: https://neuro-jena.github.io/cat/<br>SPM 12 (version 7771): https://www.fil.ion.ucl.ac.uk/spm/software/spm12/<br>Preprocessing pipeline: https://github.com/XinyangYu918/EatingBehaviours-BrainMaturation-Psychopathology-Genetics/blob/main/Preprocessing.txt<br>Preprocessing batch: https://github.com/XinyangYu918/EatingBehaviours-BrainMaturation-Psychopathology-Genetics/blob/main/batch_long_420s_1-420.m<br>Analysis scripts: https://github.com/XinyangYu918/EatingBehaviours-BrainMaturation-Psychopathology-Genetics/blob/main/Linear%20mixed%20models%20for%20CT%20and%20SD.R<br>MRIcron (version 1.0.2019): https://www.nitrc.org/projects/mricron<br>BrainNet Viewer (version: 20191031): https://www.nitrc.org/projects/bnv/<br>ENIGMA visualisation tool: https://github.com/MICA-MNI/ENIGMA/tree/master/enigmatoolbox<br><br>Software and code for the genetic analyses can be assessed at:<br>ENIGMA genotyping QC and imputation protocol: https://enigma.ini.usc.edu/wp-content/uploads/2020/02/ENIGMA-1KGP_p3v5-Cookbook_20170713.pdf<br>plink 1.9: https://www.cog-genomics.org/plink/<br>KING (version 2.3.2): https://www.kingrelatedness.com/

GENESIS R package (version 2.32.0): https://www.bioconductor.org/packages/release/bioc/html/GENESIS.html
Michigan Imputation Server using Minimac 4: https://imputationserver.sph.umich.edu/index.html#!run/minimac4
Quality control scripts prior and post imputation: https://github.com/XinyangYu918/EatingBehaviours-BrainMaturation-Psychopathology-Genetics/tree/main
PRS-CS (version May14, 2014): https://github.com/getian107/PRScs

Software and code for latent growth curve modelling analysis are available at:
lavaan R package (version 0.6-17): https://cran.r-project.org/web/packages/lavaan/index.html
Scripts for LGCM analysis: https://github.com/XinyangYu918/EatingBehaviours-BrainMaturation-Psychopathology-Genetics/blob/main/LGCM.R

Software and code for mediation analysis can be assessed at:
PROCESS macro for R: https://www.processmacro.org/index.html
AMOS: https://www.ibm.com/products/structural-equation-modeling-sem

Other software and code used in the sensitivity analysis can be found at:
jBmi R package: https://github.com/jbirstler/jBmi
Scripts for all other analyses: https://github.com/XinyangYu918/EatingBehaviours-BrainMaturation-Psychopathology-Genetics/tree/main

For manuscripts utilizing custom algorithms or software that are central to the research but not yet described in published literature, software must be made available to editors and reviewers. We strongly encourage code deposition in a community repository (e.g. GitHub). See the Nature Portfolio guidelines for submitting code & software for further information.

Data

Policy information about availability of data

All manuscripts must include a data availability statement. This statement should provide the following information, where applicable:

- Accession codes, unique identifiers, or web links for publicly available datasets
- A description of any restrictions on data availability
- For clinical datasets or third party data, please ensure that the statement adheres to our policy

Access to individual-level data from the IMAGEN project is accessible to bona fide researchers upon reasonable request and approval of a project proposal by IMAGEN consortium PIs. Contact the corresponding author for requests related to this study.
Summary statistics from the BMI GWAS, used in this study for computing BMI PGS, are accessible via57, and can be downloaded from their website at https://portals.broadinstitute.org/collaboration/giant/index.php/GIANT_consortium_data_files. Data from the 1000 Genomes Project Phase 3 may be accessed from https://www.internationalgenome.org/category/phase-3/. The 'Desikan-Killiany' cortical atlas, used in this study for cortical parcellation, is implemented in the FreeSurfer software.

Research involving human participants, their data, or biological material

Policy information about studies with human participants or human data. See also policy information about sex, gender (identity/presentation), and sexual orientation and race, ethnicity and racism.

Reporting on sex and gender	Self-reported 'sex' was used due to its biological attribution. Sex was included as covariates in all analyses. N = 943 male and N = 956 female participants were included in generating polygenic scores for body mass index. N = 478 male and N = 518 female participants were included in the main analysis (see Table 1). Sex-based analyses were not performed in this study due to a lack of statistical power.
Reporting on race, ethnicity, or other socially relevant groupings	Data analysed in this study was collected as part of IMAGEN, a longitudinal genetic x neuroimaging cohort study of adolescents recruited from eight study centres in England, Ireland, France and Germany. Information on specific ethnic categories was not collected. Given its focus on genetics, the study, aimed at identifying the genetic and neurobiological basis of individual variability in behaviours, was designed to include predominantly participants of European ancestry (White), based on their self-reports. To further account for population stratification, statistical approaches were applied to identify and exclude genetic ancestries other than European, when analysing the genetic data.
Population characteristics	Demographic and covariate relevant population characteristics for the IMAGEN study are reported in Table 1 and in the Methods section. The available sample sizes for baseline and follow-up assessments included in this study are provided in Supplementary Table S1.
Recruitment	All individuals were recruited from eight study centres in England, Ireland, France and Germany.
Ethics oversight	The IMAGEN study was approved by local research ethics committees at each study site, and informed consent was obtained from participants and their parents/guardians.

Note that full information on the approval of the study protocol must also be provided in the manuscript.

Field-specific reporting

Please select the one below that is the best fit for your research. If you are not sure, read the appropriate sections before making your selection.

☒ Life sciences ☐ Behavioural & social sciences ☐ Ecological, evolutionary & environmental sciences

Life sciences study design

All studies must disclose on these points even when the disclosure is negative.

Sample size	Data analysed in this study was collected as part of IMAGEN project. We restricted our analyses to participants who had completed the Three-Factor Eating Questionnaire (TFEQ) scores at age 23 (to assess their eating behaviours) and had at least one available measure from the Strengths and Difficulties Questionnaire (SDQ) at ages 14, 16, 19 and 23. This allowed us to perform latent growth curve modelling to compare how these participants differed in their internalising and externalising problem trajectories. . A total of 996 participants (478 male and 518 female participants) met these criteria and were included in the study. For the neuroimaging analysis, we included participants with available MRI data available at ages 14 and 23, excluding 47 participants whose data failed to meet our quality control criteria. This resulted in 949 participants being included in the MRI analysis. For polygenic scoring, we restricted our sample to participants of European ancestry, resulting in a final sample of 881 participants for the genetic analysis.<br>The sample of 996 participants met the requirements for latent growth curve modelling analysis, where a minimum sample size of 100-200 per group is recommended for reliable estimates. It also met the standards for neuroimaging studies, where 100-150 participants are generally considered sufficient to detect moderate to large effects.
Data exclusions	For genetic data, individuals with excessive missing genotypes (> 5%) or excessive heterozygosity (3 SD from the mean), and closely related individuals estimated by identity-by-state clustering and multi-dimensional scaling analysis were excluded from the anlaysis. Due to the poor portability of polygenic scores across ancestries, individuals who were outliers from the European ancestry were excluded from the polygenic scoring. Full methods for identifying genetic ancestry for participants and QC procedures can be found in the Supplementary Information and scripts used to perform quality control can be assessed at: https://github.com/XinyangYu918/EatingBehaviours-BrainMaturation-Psychopathology-Genetics<br>For neuroimaging analysis: Participants were excluded from the analysis if they had missing MRI data or failed to meet quality control criteria (N = 47; see Methods for image pre-processing and QC).
Replication	The IMAGEN is a well-phenotyped longitudinal genetic × neuroimaging cohort study of adolescents of European ancestry. To our knowledge, there is a lack of a comparable cohort with harmonised genetics, neuroimaging and behavioural data to perform independent replication of the analyses presented in this study. However, we aim to promote future research that includes more ethnically diverse samples for broader applicability.
Randomization	N/A. IMAGEN is an observational cohort that did not include randomisation.
Blinding	N/A. IMAGEN is an observational cohort that did not involve blinding.

Reporting for specific materials, systems and methods

We require information from authors about some types of materials, experimental systems and methods used in many studies. Here, indicate whether each material, system or method listed is relevant to your study. If you are not sure if a list item applies to your research, read the appropriate section before selecting a response.

Materials & experimental systems

n/a	Involved in the study	
☒	☐	Antibodies
☒	☐	Eukaryotic cell lines
☒	☐	Palaeontology and archaeology
☒	☐	Animals and other organisms
☒	☐	Clinical data
☒	☐	Dual use research of concern
☒	☐	Plants

Methods

n/a	Involved in the study	
☒	☐	ChIP-seq
☒	☐	Flow cytometry
☐	☒	MRI-based neuroimaging

Plants

Seed stocks	*Report on the source of all seed stocks or other plant material used. If applicable, state the seed stock centre and catalogue number. If plant specimens were collected from the field, describe the collection location, date and sampling procedures.*
Novel plant genotypes	*Describe the methods by which all novel plant genotypes were produced. This includes those generated by transgenic approaches, gene editing, chemical/radiation-based mutagenesis and hybridization. For transgenic lines, describe the transformation method, the number of independent lines analyzed and the generation upon which experiments were performed. For gene-edited lines, describe the editor used, the endogenous sequence targeted for editing, the targeting guide RNA sequence (if applicable) and how the editor was applied.*
Authentication	*Describe any authentication procedures for each seed stock used or novel genotype generated. Describe any experiments used to assess the effect of a mutation and, where applicable, how potential secondary effects (e.g. second site T-DNA insertions, mosiacism, off-target gene editing) were examined.*

Magnetic resonance imaging

Experimental design

Design type	Structural MRI
Design specifications	No functional MRI data was analysed in this study.
Behavioral performance measures	No functional MRI data was analysed in this study.

Acquisition

Imaging type(s)	T1-weighted structural
Field strength	3 Tesla
Sequence & imaging parameters	MRI images were acquired with 3T MRI scanners from different manufacturers (Siemens, Munich, Germany; Philips, Best, The Netherlands; General Electrics, Chalfont St Giles, UK; Bruker, Ettlingen, Germany) from eight IMAGEN recruitment sites. The high-resolution anatomical MRI images acquired included a three-dimensional T1-weighted magnetisation prepared gradient echo sequence (MPRAGE) based on the ADNI protocol (http://adni.loni.usc.edu/methods/documents/mri-protocols/), T2 weighted fast-spin echo, and FLAIR scans for visual assessment.
Area of acquisition	Whole brain

Diffusion MRI ☐ Used ☒ Not used

Preprocessing

Preprocessing software	CAT 12.8 r1907: https://neuro-jena.github.io/cat/
Normalization	Diffeomorphic Anatomical Registration Through Exponentiated Lie Algebra (DARTEL) normalisation was subsequently performed on the segmented mean images using the default DARTEL template.
Normalization template	The derived spatial normalisation parameters were then applied to transform the segmented subject baseline and follow-up grey matter images into the standard Montreal Neurological Institute (MNI) space.
Noise and artifact removal	All raw images were visually inspected to exclude images with movement artefacts, brace artefacts, or field inhomogeneities prior to pre-processing.<br>The quality measures created during pre-processing for each participant at each time point were examined, and images with sufficient quality (corresponding to grade D or above) were included in further analyses.
Volume censoring	N/A. No functional MRI data were analysed in this study.

Statistical modeling & inference

Model type and settings	For whole-brain voxel-based morphometry analysis: longitudinal whole-brain voxel-based morphometry (VBM) analyses were performed using the CAT 12.8 (r1932) toolbox. To identify brain regions reflecting significant changes in grey matter volumes (GMVs) between ages 14 and 23 among the groups identified above, we performed a 2 × 2 mixed ANOVA on the smoothed images using the "Flexible Factorial" model. The two factors were age (age 14 or age 23; within-subject) and group (i.e., comparison of each of 2 groups, namely REs versus HEs, E/UEs versus HEs, or REs versus E/UEs; between-subject).<br>For ROI-based analysis: linear mixed models were performed to investigate interactions between age and group, and included age, group, and their interactions as fixed effects, with participants nested within recruitment sites as a random effect and adjusted for sex.
Effect(s) tested	Flexible Factorial models implemented in the CAT 12.8 and linear mixed models.

Specify type of analysis: ☐ Whole brain ☐ ROI-based ☒ Both

Anatomical location(s)	The grey matter volume data were parcellated based on the automated automical (AAL) atlas.<br>Surface-based measures (cortical thickness and sulcal depth) were derived from the Desikan-Killiany atls.
Statistic type for inference (See Eklund et al. 2016)	Voxel-wise analysis and ROI-based analysis.
Correction	For whole-brain VBM analysis:a cluster-level family-wise error correction with a p-value < 0.05, and a cluster-forming threshold of p-value < 0.001 without correction.<br>For ROI-based analysis: Bonferroni correction was applied to adjust for multiple testing.

Models & analysis

n/a	Involved in the study
☒	☐ Functional and/or effective connectivity
☒	☐ Graph analysis
☐	☒ Multivariate modeling or predictive analysis

Multivariate modeling and predictive analysis

Multivariate latent growth curve modelling and multivariate mediation analyses were performed in this study.
